# METTL3 regulates Leydig cell proliferation via miR-145-PCK1 mediated gluconeogenesis in goats

**DOI:** 10.1186/s40104-025-01307-5

**Published:** 2026-01-17

**Authors:** Wen Tang, Maosheng Cao, Fengxin Qiao, Jinhong Luo, Yonghong Ju, Xiaodong Wang, Pengchen An, Wei Sun, Xiang Chen

**Affiliations:** 1 Key Laboratory of Animal Genetics, Breeding and Reproduction in The Plateau Mountainous Region, Ministry of Education, Guizhou University, Guiyang, 550025 China; 2 Key Laboratory of Animal Genetics, Breeding and Reproduction, Guiyang, 550025 China; 3 College of Animal Science, Guizhou University, Guiyang, 550025 China; 4 College of Animal Science and Technology, Yangzhou University, Yangzhou, 225009 China

**Keywords:** Leydig cells, Methyltransferase like 3, MiR-145-3p, *PCK1*, Testicular development

## Abstract

**Background:**

Normal testicular development is essential for maintaining male fertility and reproductive performance in livestock. Leydig cells (LCs) play a central role in testicular physiology; however, the epigenetic mechanisms regulating their development remain largely unclear. Methyltransferase-like 3 (METTL3), a key m^6^A methylation enzyme, and microRNAs are increasingly recognised as critical regulators of this process.

**Results:**

METTL3 expression in goat LCs markedly decreased during testicular development. This downregulation reduced m^6^A modification on pri-miR-145, impairing DiGeorge syndrome critical region 8-mediated processing and resulting in decreased levels of mature miR-145-3p. This reduction in miR-145-3p increased the expression of phosphoenolpyruvate carboxykinase 1 (*PCK1*), which activated gluconeogenesis, increased intracellular glucose levels, and increased mitochondrial membrane potential. Consequently, this metabolic shift upregulated cell cycle-related genes (cyclin B1 and cyclin E2), promoting LC proliferation and testicular growth.

**Conclusions:**

Our findings demonstrate that the METTL3/miR-145-3p/PCK1 axis is a key regulatory pathway linking epigenetic modification to the metabolic activity and proliferation of LCs. This mechanism provides novel insights into the molecular control of testicular development in male goats and may offer new targets for improving male reproductive capacity in livestock.

**Supplementary Information:**

The online version contains supplementary material available at 10.1186/s40104-025-01307-5.

## Introduction

In recent years, epigenetic modifications have been recognized as important regulators of various physiological processes and disease progressions. N^6^-methyladenosine (m^6^A) methylation is the most prevalent and abundant internal RNA modification in eukaryotic messenger RNAs (mRNAs) [1], microRNAs (miRNAs) [2], and long noncoding RNAs (lncRNAs) [3]. Its biological function is mediated by three binding proteins with different functions: methyltransferases, demethylases, and methylation-reading proteins [4]. The methyltransferase complex plays an important role in mammalian spermatogenesis, including processes such as spermatogonial stem cell differentiation, meiosis, and spermatogenesis. In mice, conditional methyltransferase-like 3 (*METTL3*) knockout in male germ cells leads to defects in spermatogenesis by modulating spermatogonial stem cell differentiation and meiosis [5], while conditional *METTL14* knockout in germ cells leads to decreased m^6^A modification, excessive proliferation, and depletion of spermatogonial stem cells, thereby disrupting spermatogenesis [6]. In *METTL3* and *METTL14* double-knockout mice, sperm exhibit severely reduced motility, flagellar defects, and sperm head deformity [6]. Knockout of *METTL3* and *METTL14* leads to dysregulation of the translation of key regulatory factors (such as *PLZF*, *ID4*, *DNMT3b*, and *SOHLH2*) in spermatogonial stem cells [6]. However, whether METTL3-mediated m^6^A modification is involved in testicular development remains unclear.

The development of biomedical research in recent years has enabled the elucidation of key mechanisms underlying reproductive system regulation. In this context, Leydig cells (LCs), which are key cells in testicular development, have attracted increasing attention for their roles in androgen synthesis and spermatogenesis regulation [7, 8]. As the main endocrine cells, LCs not only are responsible for androgen biosynthesis to maintain male reproductive function but also directly or indirectly regulate the homeostasis of seminiferous tubules by secreting various factors [9]. LC-secreted testosterone directly drives spermatogonial differentiation, meiosis, and spermatogenesis by activating androgen receptors in the seminiferous epithelium [10]. In addition to testosterone, insulin-like growth factor 1 secreted by LCs promotes energy metabolism in spermatocytes through the phosphoinositide 3-kinase (PI3K)/protein kinase B (AKT) pathway, and its gene expression is regulated by the GATA4/COUP transcription factor 2 transcriptional complex [11]. Moderate LC proliferation is important for maintaining normal serum testosterone levels, which are closely related to reproductive capacity. In contrast, dysfunction of LCs leads to failure of spermatogenesis, which often manifests as azoospermia [12]. Metabolic abnormalities in LCs lead to an imbalance in the expression of *INSL3* and androgen receptors, further causing spermatogenic disorders [13]. As previous studies have mostly focused on the mechanisms of LCs in androgen synthesis, the specific roles and regulatory mechanisms of LCs in glucose metabolism remain poorly defined [14, 15].

The dynamic balance of energy metabolism during testicular development has a decisive effect on the establishment of spermatogenic function. Because the testis has low oxygen content under normal conditions, spermatogenic cells mainly utilize lactate produced by glycolysis in Sertoli cells as an energy substrate [16]. As the testis develops, the glycolytic activity of Sertoli cells and the lactate levels increase. Knockdown of the lactate dehydrogenase A gene directly leads to spermatogenic arrest, confirming that lactate is not only an energy substrate but also involved in regulating testis development [17]. Theoretically, gluconeogenesis is the reverse of glycolysis, which converts non-carbohydrate carbon sources, such as lactic acid, amino acids, and triglycerides, into glucose to meet energy needs. Gluconeogenesis synthesizes normal components of the cell membrane (such as glycolipids, glycoproteins, and structural polysaccharides) by producing glucose derivatives. It also processes lactic acid produced by glycolysis, thereby alleviating metabolic acidosis. We speculate that during testicular development, interstitial cells may activate the gluconeogenic pathway to promote glucose synthesis and, subsequently, testicular development.

Although studies have preliminarily confirmed that *METTL3* regulates LCs, the specific regulatory mechanisms and functions of *METTL3* remain unclear. Here, we demonstrate that the expression of *METTL3* gradually decreases during goat testicular development, accompanied by a downregulation of mature miRNAs and an upregulation of gluconeogenesis-related genes. Mechanistically, we reveal that in LCs, METTL3 modulates the m^6^A modification level of miRNA-145, leading to a decrease in miRNA-145-3p levels. This downregulation promotes the expression of the gluconeogenesis gene *PCK1*, ultimately stimulating the proliferation of LCs. This study reveals a new level of the regulatory network of testicular development, providing an important scientific basis.

## Materials and methods

### Test animals and sample collection

The experiments were conducted following the ethical guidelines set by the Ethics Committee of Guizhou University in compliance with animal welfare regulations (EAE-62 U-2022-T055). In this study, we selected healthy Qianbei Ma goats with detailed genealogical records and divided them into two groups according to age (0 and 6 months), each comprising five rams. The test animals were provided by Xishui Fuxing Husbandry Co., Ltd. (Zunyi, Guizhou, China). Testicular samples were collected from goats on the same day. Initially, the goats were anesthetized using standard veterinary procedures, followed by immediate castration. The left testicular tissue was obtained and rinsed with phosphate-buffered saline within 20 min of excision. After collection, the testicular tissue was subdivided for different analyses: one portion was fixed in 4% paraformaldehyde, another portion was rapidly frozen in liquid nitrogen and stored at −80 °C for sequencing analysis, and the remaining fresh tissue was immediately processed for the isolation and culture of primary Leydig cells.

### Histological and ultrastructural analysis

Testicular tissues were fixed in 4% paraformaldehyde overnight at room temperature, embedded in paraffin, and sectioned at a thickness of 5 µm. Hematoxylin and eosin (HE) staining was performed to evaluate the overall histological architecture, and sections were examined under an Olympus fluorescence microscope (IX71; Olympus, Tokyo, Japan). For immunohistochemical analysis, sections were treated with 3% hydrogen peroxide for 10 min to quench endogenous peroxidase activity, followed by incubation with a primary antibody against METTL3 (1:200, ab195352, Abcam) overnight at 4 °C. After washing, the sections were incubated with a secondary antibody (1:500 in blocking solution) for 1 h, and the signal was visualized using 3,3′-diaminobenzidine (DAB; JKGREEN). Images were acquired using an Olympus imaging suite (cellSens Dimension; Olympus). For scanning electron microscopy (SEM), testicular samples were fixed in 2.5% glutaraldehyde at 4 °C overnight, rinsed with 0.1 mol/L phosphate buffer (pH 7.4), and post-fixed with 1% osmium tetroxide for 1 h. Samples were dehydrated through a graded ethanol series, replaced with isoamyl acetate, dried using a critical point dryer, mounted on aluminum stubs, and sputter-coated with gold. SEM images were obtained using a Apero 2 (Thermo Fisher Scientific) instrument at an accelerating voltage of 5–10 kV.

### Isolation and culture of testicular LCs

According to a previously published method [18], the specific isolation and culture of testicular LCs were as follows. First, the testicular interstitial tissue was stripped, and the testicular tissue was cut into 1 mm^3^ pieces under aseptic conditions on a clean bench via operation. Subsequently, the testicular tissue pieces were digested with trypsin for 8 min. Dulbecco’s modified eagle medium/F12 containing 1% (v/v) penicillin/streptomycin (Invitrogen, CA, USA) and 10% fetal bovine serum (FBS; HyClone, USA) was added as a complete culture medium to terminate digestion. The digested solution containing the separated cells was filtered through 250- and 400-mesh to collect the cells. Finally, the collected cells were cultured to 80% confluence in a cell culture box and the culture medium was regularly replaced.

### Cellular immunofluorescence

Goat LCs were cultured in 12-well optical bottom plates. The cells were washed thrice with phosphate-buffered saline (PBS), immersed in ice-cold methanol for 20 min, and then rinsed with PBS. The cells were permeabilised with 0.1% Triton X-100 for 10 min and then blocked with 1% goat serum for 30 min. They were then incubated at 4 °C for 12 h with anti-CYP17A1 (1:100; Proteintech, Wuhan, China) primary antibodies. The cells were washed thrice with PBS and incubated with fluorescein-conjugated immunoglobulin G (IgG) antibodies (1:200; Proteintech). After 1 h, the cells were washed thrice with PBS and then stained with 4′,6-diamidino-2-phenylindole solution (1:1,000; Beyotime, China) for 10 min. Finally, LCs were observed using an Olympus fluorescence microscope (IX71; Olympus, Tokyo, Japan), and images were obtained using an Olympus application suite (cellSens Dimension; Olympus). The images (Fig. S1; merge; bottom row) represent the merged fluorescence channels. Goat LCs were stained green for FSHR and blue for nuclei.

### Cell treatment and transfection

LCs were seeded in 12- or 6-well plates at a density of 4 × 10^5^ cells/well or 1 × 10^6^ cells/well, respectively, until they reached 70% confluency. *METTL3* siRNA (100 nmol/L) or siNC (100 nmol/L) were transfected into the cells using a Lipofectamine™ 3000 transfection reagent (Invitrogen, MA, USA) following the manufacturer’s protocol. To determine whether *METTL3* induces proliferation through the gluconeogenic pathway, a 10 μmol/L dose of the gluconeogenic inhibitor 3-MPA (MCE, UK) was added 12 h after transfection along with siMETTL3, forming the siMETTL3 + 3-MPA group. Although 3-MPA is widely used as a PCK1 inhibitor, its specific is limited and it may also inhibit PCK2 or other metabolic enzymes under certain conditions; this potential off-target effect should be considered when interpreting the results.

To determine whether miR-145-3p inhibition or overexpression promoted PCK1 expression, the cells were transfected with miR-145-3p mimics, mimic-NC (100 nmol/L), inhibitor (100 nmol/L), or inhibitor-NC (100 nmol/L) or miR-145-3p using a Lipofectamine™ 3000 transfection reagent (Invitrogen, CA, USA). Whether miR-145-3p induces proliferation via the gluconeogenesis pathway was determined by adding 10 μmol/L of 3-MPA at 12 h of transfection along with mimics-miR-145-3p, forming the mimics-miR-145-3p + 3-MPA group. All siRNAs (GeneBiogist, China) are presented in Table S1.

To investigate the mechanism of PCK1-induced proliferation in LCs, the OE-PCK1 plasmid was cloned using pcDNA3.1(+) as the vector, and the pcDNA3.1-GFP null plasmid was used as the negative modulate (OE-NC; GeneBiogist). The OE-PCK1 (2 μg/mL) or OE-NC (2 μg/mL) plasmid was transfected into cells using a Lipofectamine™ 3000 transfection reagent (Invitrogen, CA, USA), following the manufacturer’s protocol. We determined whether *PCK1* induces proliferation via the gluconeogenesis pathway by adding 10 μmol/L of 3-MPA at 12 h of transfection along with OE-PCK1, forming the OE-PCK1 + 3-MPA group.

### Luciferase activity assay

The luciferase activity assay was performed as previously described [19]. The constructed plasmids were co-transfected with miR-145-3p. HEK293 cells were collected 24 h after transfection. The luciferase assay was performed according to the manufacturer’s instructions (Promega, Madison, WI, USA).

### MeRIP assay

*METTL3* was silenced in goat LCs using siRNA lysed with RIPA buffer (Solarbio, Beijing, China) supplemented with proteinase inhibitor cocktail (MCE, UK) at 4 °C. Incubation with an anti-DGCR8 antibody (1:1,000, Abcam, UK) overnight at 4 °C was performed for the immunoprecipitation of endogenous DGCR8. RNA was extracted with phenol:chloroform:isoamyl alcohol (25:24:1) and detected via quantitative real-time reverse transcription PCR (qRT-PCR) using specific primers, followed by normalization to the input value.

For the m^6^A RNA-binding assay, the level of miR-145-3p was detected using a MeRIP kit (A-P-9018, IVDSHOW, China) according to the manufacturer's protocol. Briefly, METTL3 was silenced in goat LCs using a siRNA. Cellular RNA was extracted 24 h after transfection. Immunoprecipitation was performed using an m^6^A antibody (A-P-9018, IVDSHOW) previously conjugated with magnetic Dynabeads (A-P-91, IVDSHOW) in RIPA buffer and incubated with RNA without DNA. Then, the beads were treated with proteinase K at 55 °C for 15 min. RNA was extracted with phenol:chloroform:isoyl alcohol (25:24:1) and detected via qRT-PCR using pri-miR-145 primers, followed by normalization to the input data.

### Co-immunoprecipitation assay

*METTL3* was silenced in goat LCs using a siRNA and lysed with RIPA buffer (Solarbio) at 4 °C, supplemented with a protease inhibitor cocktail (MCE). Cell extracts were immunoprecipitated with anti-METTL3 antibody protein A/G magnetic beads (HY-K0202, MCE) overnight at 4 °C. After washing, the immunoprecipitated complexes were incubated with either RNase A or RNase inhibitor (R1030, Solarbio) for 5 min at 37 °C. Protein blotting was then performed using anti-DGCR8 (1:1,000; Abcam).

### Cell viability assay

Cell viability was measured using a Cell Counting Kit-8 (CCK8; Solarbio) according to the manufacturer’s protocol. LCs were cultured in a 96-well plate, with each well containing approximately 1 × 10^4^ cells in 10 µL of cell culture medium. After 24 h, the culture medium was replaced with pre-warmed, fresh, serum-free culture medium. Subsequently, the LCs were transfected with siMETTL3, siMETTL3 + 3-MPA, mimics-miR-145-3p, inhibitor-miR-145-3p, mimics-miR-145-3p + 3-MPA, OE-PCK1 siPCK1, or OE-PCK1 + 3-MPA for 24 h in fresh medium. Then, 10 μL of CCK8 solution was added to each well and incubated for 2 h. The cells were quantified using an ELX 800 Universal Microplate Reader (BioTek, USA) and the absorbance was measured at 450 nm (OD_450_) using a spectrophotometer.

### Measurement of mitochondrial membrane potential level

Mitochondrial membrane potential was assessed using a JC-1 fluorescent probe (Beyotime, China). After treatment, LCs were incubated with JC-1 according to the manufacturer’s instructions. Fluorescence signals were acquired under a fluorescence microscope by detecting JC-1 aggregates (red fluorescence, excitation/emission: 525/590 nm) and JC-1 monomers (green fluorescence, excitation/emission: 490/530 nm). The mitochondrial membrane potential was quantified by calculating the ratio of red to green fluorescence intensity.

### Glucose uptake assay

Glucose uptake in LCs was analyzed using the 2-(N-(7-nitrobenz-2-oxa-1,3-diazol-4-yl) amino (2-NBDG; Life Technologies, Carlsbad, CA, USA) method as previously described [19]. Briefly, LCs were seeded in a 12-well plate, starved for 2 h, and subjected to different treatments in serum-free medium for 24 h. The medium was discarded, after which the cells were washed with phosphate-buffered saline thrice and treated with 2-NBDG (100 µmol/L). After 1 h, the cells were collected and analyzed using fluorescence microscopy (IX71; Olympus).

### RNA extraction, library construction, and sequencing

Otal RNA was extracted from testicular tissues of the 0-month and 6-month groups using TRIzol reagent (Invitrogen, CA, USA) following the manufacturer’s protocol, which included tissue homogenisation, chloroform phase separation, isopropanol precipitation, ethanol washing, and resuspension in RNase-free water. DNase I treatment was applied to remove genomic DNA contamination. RNA quantity and purity were measured using a NanoDrop 2000 (Thermo Fisher Scientific, MA, USA), and RNA integrity was evaluated using an Agilent 4200 TapeStation (Agilent Technologies, CA, USA); only samples with RNA integrity number ≥ 7.0 were used for library preparation. Libraries were constructed using a NEBNext® Ultra™ II RNA Library Prep Kit for Illumina (New England Biolabs, MA, USA) according to the manufacturer’s instructions. Sequencing was performed on an Illumina NovaSeq 6000 platform with 2 × 150 bp paired-end reads and an average depth of ~ 30 million reads per sample. Raw reads were quality-filtered to remove adapters and low-quality sequences and mapped to the reference genome, and transcript-level expression was quantified using StringTie. Differential expression analysis was performed using edgeR, defining significantly different mRNAs as log_2_ (fold change) ≥ 1 and *P* < 0.05. The raw sequencing data are available at NCBI BioProject PRJNA917820 (https://www.ncbi.nlm.nih.gov/bioproject/PRJNA917820).

### Gene Ontology (GO) and Kyoto Encyclopedia of Genes and Genomes (KEGG) pathway analysis

Differentially expressed genes were subjected to functional enrichment analysis using the Gene Ontology (GO) database (http://www.geneontology.org) and the Kyoto Encyclopedia of Genes and Genomes (KEGG) database (http://www.genome.jp/kegg). GO enrichment analysis included three categories: biological process (BP), molecular function (MF), and cellular component (CC). KEGG pathway enrichment analysis was performed to identify significantly enriched signalling and metabolic pathways. Both analyses were conducted using the DAVID 6.8, and enrichment results were considered statistically significant at an adjusted *P* value (false discovery rate, FDR) < 0.05.

### RNA extraction and qRT-PCR

Total RNA was extracted using an RNA isolation kit (Sigma-Aldrich, UK) and reverse-transcribed to cDNA using Prime Script RT and gDNA removal kits (Abm, Canada). For the qRT-PCR reaction, a total volume of 20 μL was prepared, consisting of 10 μL SYBR (Abm, Canada), 0.5 μmol/L forward primer, 0.5 μmol/L reverse primer, 5 ng/μL cDNA, and DEPC water. qRT-PCR was performed using the following protocol: 95 °C for 2 min, followed by 50 cycles of 95 °C for 15 s and 60 °C for 30 s. Data were analyzed using the comparative 2^−△△Ct^ method, with β-actin as the housekeeping gene. The primers used were synthesized by Tsingke Biotech Co., Ltd. (Beijing, China) (Table S2).

### Protein extraction and Western blotting

RIPA lysis buffer (Solarbio, Beijing, China) supplemented with a protease inhibitor cocktail was used to lyse testicular tissues and cultured cells for protein isolation. The lysates were centrifuged at 12,000 × *g* for 10 min at 4 °C to harvest cellular proteins. Protein concentrations were measured using a bicinchoninic acid protein assay kit (Beyotime, China). Total proteins were separated via 12% sodium dodecyl sulfate–polyacrylamide gel electrophoresis and transferred onto a polyvinylidene difluoride membrane (0.2 μmol/L; Millipore, USA) using a semi-wet transfer system (Bio-Rad; Hercules, CA, USA). The membrane was blocked with 5% bovine serum albumin (Sigma-Aldrich, UK) for 1 h and incubated with primary antibodies overnight at 4 °C. The membrane was washed with TBST thrice at 5 min intervals and was then incubated with horseradish peroxidase-conjugated goat anti-rabbit secondary antibody (1:10,000, Proteintech, Wuhan, China) for 1 h at room temperature. The membrane was washed with TBST thrice at 5 min intervals. Proteins were detected using the BeyoECL Star Kit (Yamei, China), and relative protein levels were analyzed by grayscale scanning using the Tanon gel imaging system (Tanon, Shanghai, China). Detailed information regarding the antibodies used for Western blotting is provided in Table S3.

### EdU standing

Testicular cells were cultured to the desired density and labeled with EdU (Beyotime, China). After fixation and membrane permeabilization, EdU incorporation was detected using the click chemistry reaction provided in the EdU assay kit, and nuclei were counterstained with DAPI. Fluorescent signals were observed under a fluorescence microscope, and the proportion of EdU-positive cells was quantified using image analysis software to assess cellular proliferation.

## Statistical analysis

All data are presented as the mean ± standard deviation (SD). Statistical analyses were performed using SPSS 19.0 software (IBM, Armonk, NY, USA). Normality of each dataset was assessed using Shapiro–Wilk test, and homogeneity of variance was evaluated using Levene’s test. For datasets meeting these assumptions, two-tailed Student's *t*-test was used for comparisons between two groups and one-way analysis of variance was used for comparisons among multiple groups. For datasets that did not satisfy these assumptions, appropriate non-parametric tests were applied as follows: Mann–Whitney U test for two-group comparisons and Kruskal–Wallis test for multiple-group comparisons. Data represent three independent experiments. Results were considered statistically significant and highly significant at *P* < 0.05 and < 0.01, respectively.

## Results

### *METTL3* gene expression decreases and *PCK1* gene expression increases in conjunction with testicular development

**Fig. 1:**
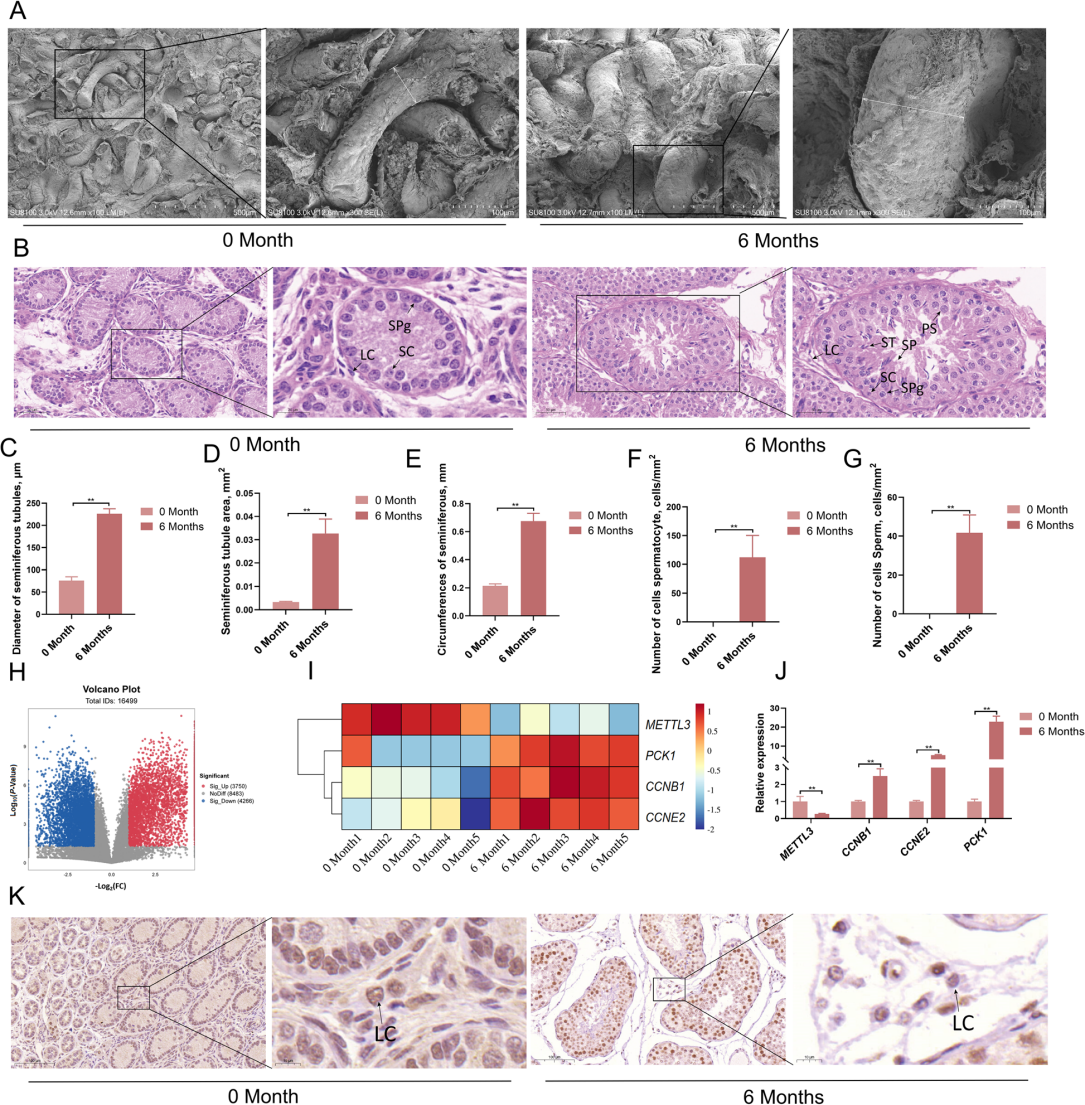
*METTL3* gene expression decreases and *PCK1* gene expression increases in conjunction with testicular development. **A** SEM analysis of testicular tissues of 0 month and 6 months Qianbei Ma goats (*n*= 5). **B** Hematoxylin and eosin staining of testicular tissues of 0 month and 6 months Qianbei Ma goats (*n*= 5). **C**–**G** Diameter of the seminiferous tubules (**C**), seminiferous tubule area (**D**), circumferences of seminiferous (**E**), number of cells spermatocyte (**F**), number of sperm cells (**G**) in Qianbei Ma goats of different ages (*n* = 5). **H** Differential gene volcanic map (*n*= 5). **I** Heat map of differential gene expression in testes of the 0 month and 6 months groups (*n *= 5). **J** The relative mRNA expression levels of *METTL3*, *CCNB1*, *CCNE2* and *PCK1* were detected by RT-qPCR in the 0 month and 6 months groups (*n *= 5). **K** The expression of METTL3 in the testis was evaluated via immunohistochemistry in the 0 month and 6 months groups (*n*= 5). Scale bar: 100 μm. **P < 0.01

Hematoxylin and eosin staining of testes showed that compared with testes collected at 0 month of age, testes at 6 months of age had rapidly growing interstitial seminiferous tubules, with a sharp increase in diameter and a rapid increase in circumference and area (Fig. 1A). Further analysis through HE staining showed that only supporting cells and spermatogonia were present in the testes at 0 month of age whereas a large number of sperm cells were visible in the seminiferous tubules at 6 months of age (Fig. 1B). Statistical analysis showed that the diameter, area, and circumference of the seminiferous tubules at 6 months of age were significantly higher than those at 0 month of age (Fig. 1C–E). Further examination of the number of spermatocytes and sperm cells revealed no spermatocytes or sperm cells in the testes at 0 month of age and a large number of sperm cells in the testes at 6 months of age (Fig. 1F–G). Transcriptomic analysis was performed on the testicular tissues of Qianbei Ma goats of different ages (Fig. 1H–I). The results revealed 3,750 upregulated and 4,266 downregulated genes in the 6-month-old group in comparison to the 0-month-old group (Fig. 1H). KEGG pathway analysis revealed that the upregulated genes were mainly enriched in the metabolic, glucose synthesis/glycolysis, and AMPK pathways (Fig. S2A). Further research showed that in 6-month-old testes, *METTL3* was downregulated whereas *PCK1* and proliferation genes (*CCNB1* and *CCNE2*) were upregulated (Fig. 1I). RT-qPCR was used to detect *METTL3* and *PCK1* expression in the testes of Qianbei Ma goats at different developmental stages. The results showed that as the testes developed, the expression of *METTL3* decreased whereas that of *PCK1*, *CCNB1*, and *CCNE2* increased (Fig. 1J). Immunohistochemical detection of METTL3 in the testes (Fig. 1K and Fig. S3) showed a decrease in METTL3 levels as the testes developed. These results indicate that with testicular development, the expression of *METTL3* in LCs decreases whereas that of the gluconeogenesis gene *PCK1* and proliferation-related genes *CCNB1* and *CCNE2* increases.

### METTL3 regulates goat LC proliferation through the gluconeogenesis pathway

**Fig. 2:**
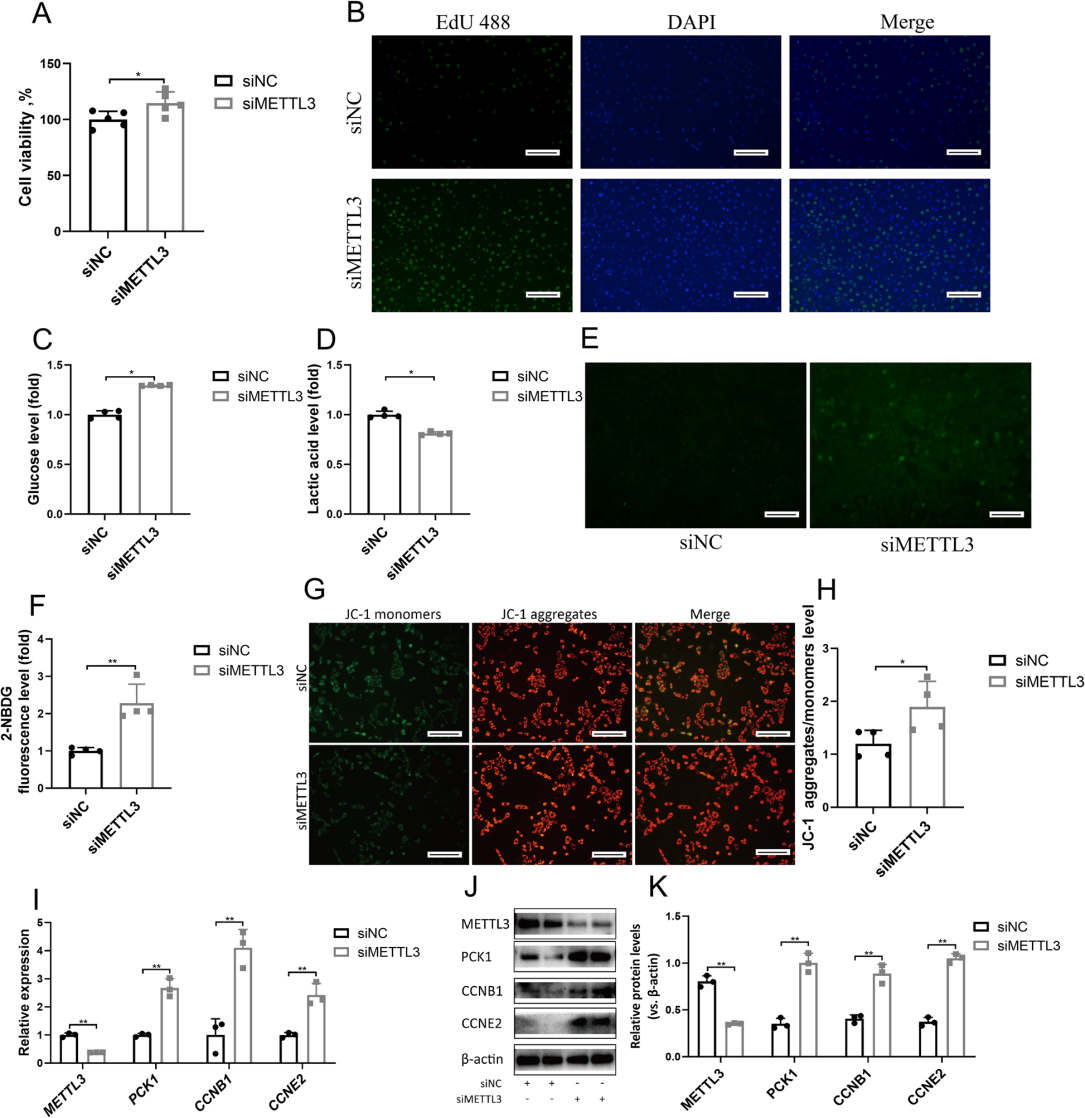
METTL3 regulates goat LC proliferation. **A** Cell viability of LCs treated with siNC and siMETTL3 for 24 h (*n* = 5). **B** LCs were treated with siNC and siMETTL3 for 24 h, and EdU (green) was used to label the proliferating cells. The cell nuclei were counterstained with DAPI (*n* = 4). Scale bar: 100 μm. **C** Glucose level of LCs treated with siNC and siMETTL3 for 24 h (*n* = 4). **D** Lactic acid level of LCs treated with siNC and siMETTL3 for 24 h (*n* = 4). **E**–**F** Glucose uptake ability of LCs treated with siNC and siMETTL3 for 24 h, assessed using immunofluorescence and quantitative analysis (*n* = 4). Scale bar: 100 μm. **G**–**H** Changes in mitochondrial membrane potential in LCs treated with siNC and siMETTL3 for 24 h were analyzed by JC-1 staining and the fluorescence intensity of JC-1 aggregates/monomers was quantified (*n* = 4). Scale bar: 100 μm. **I** The relative mRNA expression levels of *METTL3*, *PCK1*, *CCNB1* and *CCNE2* treated with siNC and siMETTL3 for 24 h (*n* = 3). **J**–**K** The levels of METTL3, PCK1, CCNB1, CCNE2 and β-actin were assessed via Western blotting. Relative protein levels were analysed via gray scanning (*n* = 3). *P < 0.05, **P < 0.01

We comprehensively explored the effects of decreased *METTL3* expression on the gluconeogenesis and proliferation of LCs during testicular development by transfecting LCs with silencing RNA (siRNA; hereafter referred to as siMETTL3). Cell Counting Kit-8 assay showed that siMETTL3 knockdown significantly increased cell viability (Fig. 2A), and EdU staining showed that the LC proliferation rate in the siMETTL3 group was significantly higher than that in the modulate group (Fig. 2B). We measured glucose levels in the siMETTL3 group to determine the potential mechanism by which siMETTL3 promotes LC proliferation. As shown in Fig. 2C–D, glucose levels increased in the siMETTL3 group, whereas lactic acid levels decreased. Further analysis revealed increased glucose uptake by LCs in the siMETTL3 group (Fig. 2E–F). An increase in energy is closely related to mitochondrial levels. In this study, the mitochondrial membrane potential levels of LCs in the siMETTL3 group significantly increased (Fig. 2G–H). Expression analysis showed that siMETTL3 significantly increased the mRNA levels of the gluconeogenesis gene *PCK1* and cell proliferation-related genes *CCNB1* and *CCNE2* in LCs (Fig. 2I), consistent with the protein levels detected (Fig. 2J–K).

**Fig. 3:**
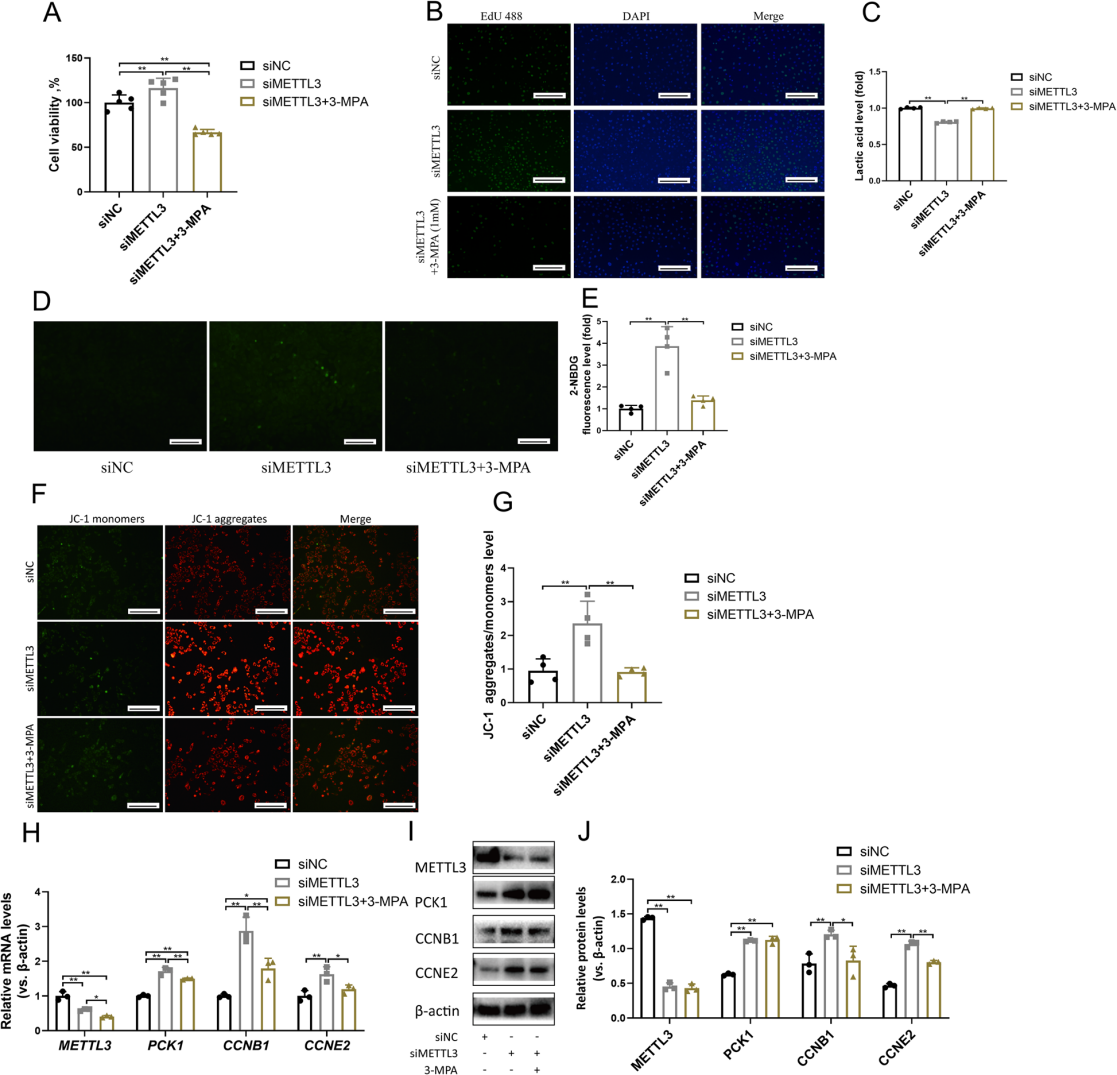
METTL3 regulates goat LC proliferation through the glyconeogenesis pathway. **A** Cell viability of LCs treated with siMETTL3 and siMETTL3+3-MPA group (*n* = 5). **B** LCs were treated with siMETTL3 and siMETTL3+3-MPA, and EdU (green) was used to label the proliferating cells. The cell nuclei were counterstained with DAPI (*n* = 4). Scale bar: 100 μm. **C** Lactic acid level of LCs treated with siNC and siMETTL3+3-MPA for 24 h (*n* = 4). **D** Glucose level of LCs treated with siMETTL3 and siMETTL3+3-MPA for 24 h (*n* = 4). **E** Glucose uptake ability of LCs treated with siMETTL3 and siMETTL3+3-MPA, assessed using immunofluorescence and quantitative analysis (*n* = 4). Scale bar: 100 μm. **F**–**G** Changes in mitochondrial membrane potential in LCs treated with siMETTL3 and siMETTL3+3-MPA were analyzed by JC-1 staining and the fluorescence intensity of JC-1 aggregates/monomers was quantified (*n* = 4). Scale bar: 100 μm. **H** The relative mRNA expression levels of *METTL3*, *PCK1*, *CCNB1* and *CCNE2* treated with siMETTL3 and siMETTL3+3-MPA (*n* = 3). **I**–**J** The levels of METTL3, PCK1, CCNB1, CCNE2 and β-actin were assessed via Western blotting. Relative protein levels were analysed via gray scanning (*n* = 3). *P < 0.05, **P < 0.01

To further investigate how METTL3 regulates the proliferation of goat LCs through the PCK1-regulated gluconeogenesis pathway, LCs were treated with the PCK1 inhibitor 3-MPA. As shown in Fig. 3A–G, 3-MPA treatment inhibited gluconeogenesis in LCs, leading to reduced proliferation, glucose uptake, mitochondrial activity, and expression of proliferation-related genes, as well as increased lactic acid levels, without affecting PCK1 expression at the mRNA and protein level (Fig. 3H–J). These results indicate that a decrease in METTL3 expression promotes PCK1 activity, which in turn enhances gluconeogenesis in and proliferation of LCs.

### Mature miRNA expression decreases in conjunction with testicular development

**Fig. 4:**
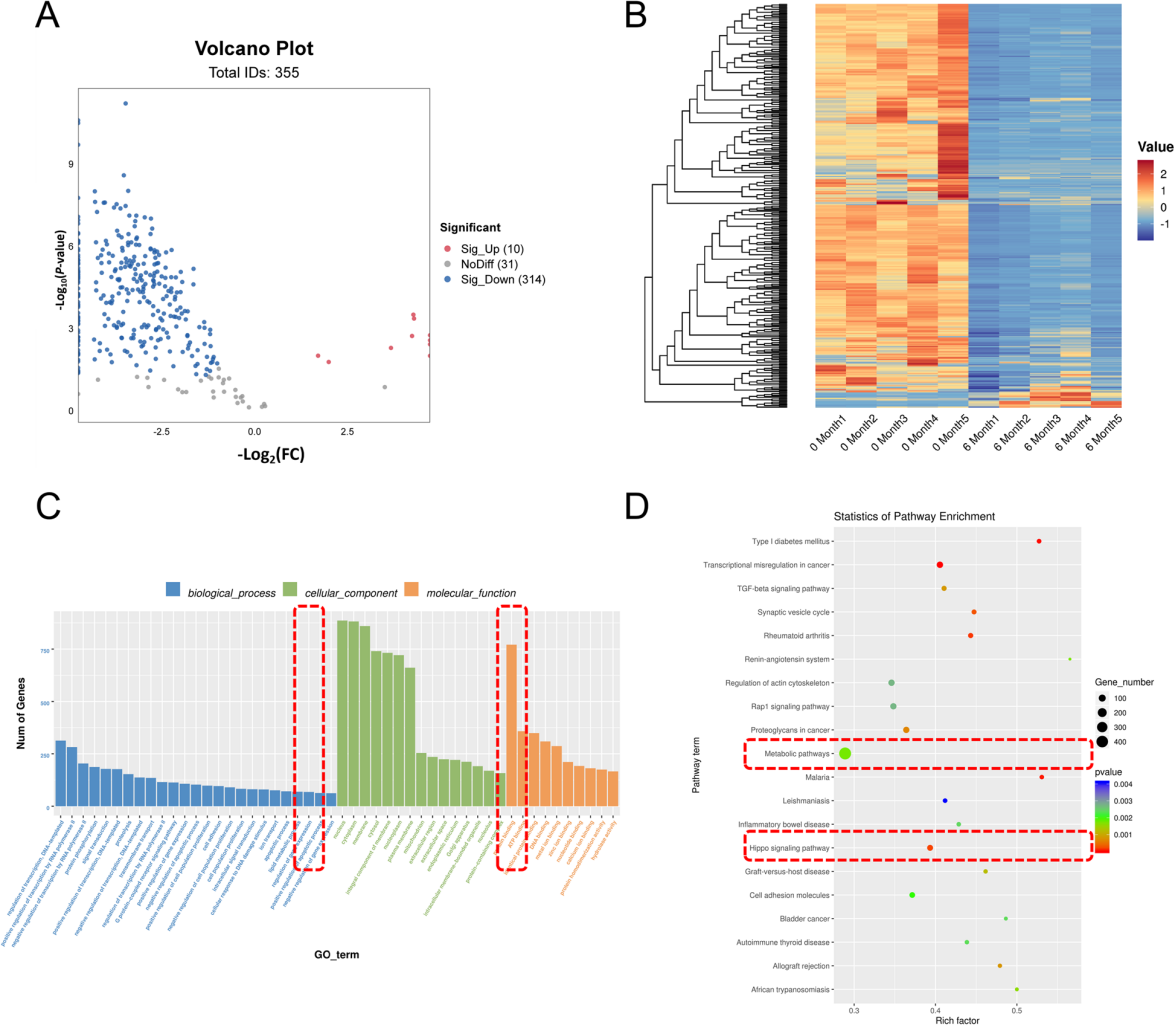
Mature miRNA expression decreases in conjunction with testicular development. **A** Differential miRNAs volcanic map (*n* = 5). **B** Heat map of differential miRNAs expression in testis of the 0 month and 6 months groups (*n* = 5). **C** GO pathway enrichment analysis of differentially down-regulated miRNA target genes (*n* = 5). **D** KEGG pathway enrichment analysis of differentially down-regulated miRNA target genes (*n* = 5)

Our previous study showed that METTL3 interacts with the RNA-binding protein DGCR8 and positively regulates the pri-miRNA process [2]. Therefore, we speculated that the decrease in METTL3 expression during testicular development leads to a reduction in mature miRNA synthesis in the testes. To verify this, we conducted an miRNA omics analysis of the testicular tissues of goats of different ages. Compared with the 0-month-old group, the 6-month-old group had 10 upregulated and 314 downregulated miRNAs (Fig. 4A–B). The target genes of the downregulated miRNAs were subjected to pathway enrichment analysis. Gene Ontology enrichment analysis revealed that the target genes of the miRNAs were closely related to ATP energy synthesis and cell proliferation (Fig. 4C). KEGG enrichment analysis revealed that the target genes of the miRNAs were closely related to metabolic pathways and the Hippo pathway (glycolysis pathway) (Fig. 4D). These results indicate that as the testes develop, the expression levels of mature miRNAs decrease.

### METTL3 regulates miR-145-3p maturation and promotes *PCK1* expression through DGCR8

**Fig. 5:**
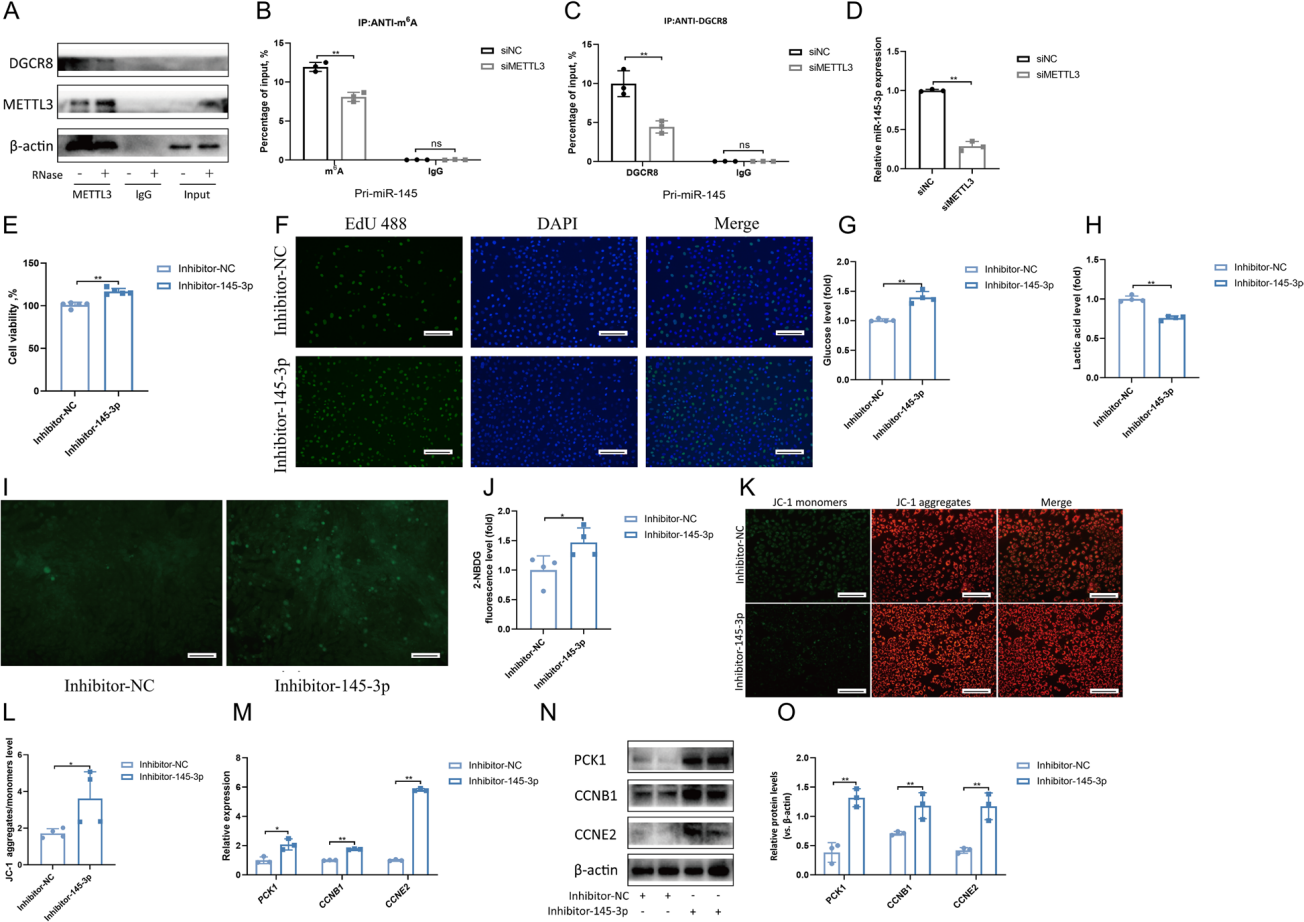
METTL3 regulates miR-145-3p maturation and promotes PCK1 expression through DGCR8. **A** Co-immunoprecipitation of the METTL3-interacting protein DGCR8. Western blotting was performed using antibodies for DGCR8 and METTL3 proteins, with IgG serving as a control for immunoprecipitation. **B** The detection of pri-miR-145 m^6^A modification level by immunoprecipitation of m^6^A modified miRNA from siNC and siMETTL3 groups in LCs followed by qRT-PCR (*n* = 3). **C** The detection of pri-miR-145 binding to DGCR8 by immunoprecipitation of DGCR8-associated RNA from siNC and siMETTL3 groups in LCs followed by qRT-PCR (*n* = 3). **D** Expression levels of miR-145-3p was detected by qRT-PCR analysis 24 h after transfection of LCs with siNC and siMETTL3 groups (*n* = 3). **E** Cell viability of LCs treated with inhibitor-NC and inhibitor-145-3p group (*n* = 5). **F** LCs were treated with inhibitor-NC and inhibitor-145-3p, and EdU (green) was used to label the proliferating cells. The cell nuclei were counterstained with DAPI (*n* = 4). Scale bar: 100 μm. **G** Glucose level of LCs treated with inhibitor-NC and inhibitor-145-3p (*n* = 4). **H** Lactic acid level of LCs treated with inhibitor-NC and inhibitor-145-3p (*n* = 4). **I–J** Glucose uptake ability of LCs treated with inhibitor-NC and inhibitor-145-3p, assessed using immunofluorescence and quantitative analysis (*n *= 4). Scale bar: 100 μm. **K**–**L** Changes in mitochondrial membrane potential in LCs treated with inhibitor-NC and inhibitor-145-3p were analyzed by JC-1 staining and the fluorescence intensity of JC-1 aggregates/monomers was quantified (*n* = 4). Scale bar: 100 μm. **M** The relative mRNA expression levels of *PCK1*, *CCNB1* and *CCNE2* treated with inhibitor-NC and inhibitor-145-3p (*n* = 3). **N**–**O** The levels of PCK1, CCNB1, CCNE2 and β-actin were assessed via Western blotting. Relative protein levels were analysed via gray scanning (*n* = 3). *P < 0.05, **P < 0.01

Further exploring the molecular mechanism by which *METTL3* downregulation in LCs promotes *PCK1* expression, we performed bioinformatics analysis on the downregulated miRNAs and *PCK1*. The results showed that miR-145-3p targets PCK1 (Fig. S4A). Luciferase reporter assay confirmed the direct binding of miR-145-3p to *PCK1* (Fig. S4B), indicating that *PCK1* is a direct target gene of miR-145-3p. Our previous study showed that *METTL3* interacts with DGCR8 and positively regulates the pri-miRNA process [2]. Therefore, this study evaluated whether the binding of DGCR8 to pri-miRNAs in LCs requires *METTL3*. The immunoprecipitation assay results showed that METTL3 binds to DGCR8 (Fig. 5A) and that this binding weakened upon RNase treatment, indicating that this binding may be partially mediated by miRNAs (Fig. 5A). The m^6^A RNA immunoprecipitation (MeRIP) assay revealed that siMETTL3 significantly reduced the amount of pri-miR-145 modified by m^6^A (Fig. 5B). Moreover, we observed decreased pri-miR-145 binding by DGCR8 immunoprecipitated from siMETTL3 LCs (Fig. 5C). Further examination of the changes in the expression of miR-145-3p in LCs after siMETTL3 treatment showed that miR-145-3p expression was significantly reduced in the siMETTL3 group (Fig. 5D). To further verify that the increase in PCK1 after siMETTL3 treatment in LCs was caused by a decrease in miR-145-3p levels, we inhibited the expression of miR-145-3p in LCs, which promoted LC proliferation (Fig. 5E–F). To explore the potential mechanism by which miR-145-3p promotes LC proliferation, we measured the glucose levels in the inhibitor-miR-145-3p group, and the results are shown in Fig. 5G–H. Glucose levels increased in the inhibitor-miR-145-3p group, while lactic acid levels decreased. Further detection of glucose uptake and mitochondrial membrane potential levels in LCs revealed an increase in glucose uptake and mitochondrial levels (Fig. 5I–L) in the inhibitor-miR-145-3p group. Furthermore, changes in the expression of *PCK1* and proliferation-related genes, along with their corresponding protein levels, were assessed (Fig. 5M–O). Inhibition of miR-145-3p significantly promoted the expression of *PCK1* and the proliferation genes *CCNB1* and *CCNE2*, and the same conclusion was drawn at the protein level. These data indicate that the reduction in *METTL3* expression in LCs leads to a decreased m^6^A modification level of pri-miR-145, further reducing the recognition and binding of the DGCR8 protein to pri-miR-145, which results in decreased synthesis of mature miR-145-3p. miR-145-3p promotes an increase in *PCK1* expression, further promoting LC proliferation.

### miR-145-3p regulates goat LC proliferation through the gluconeogenesis pathway

**Fig. 6:**
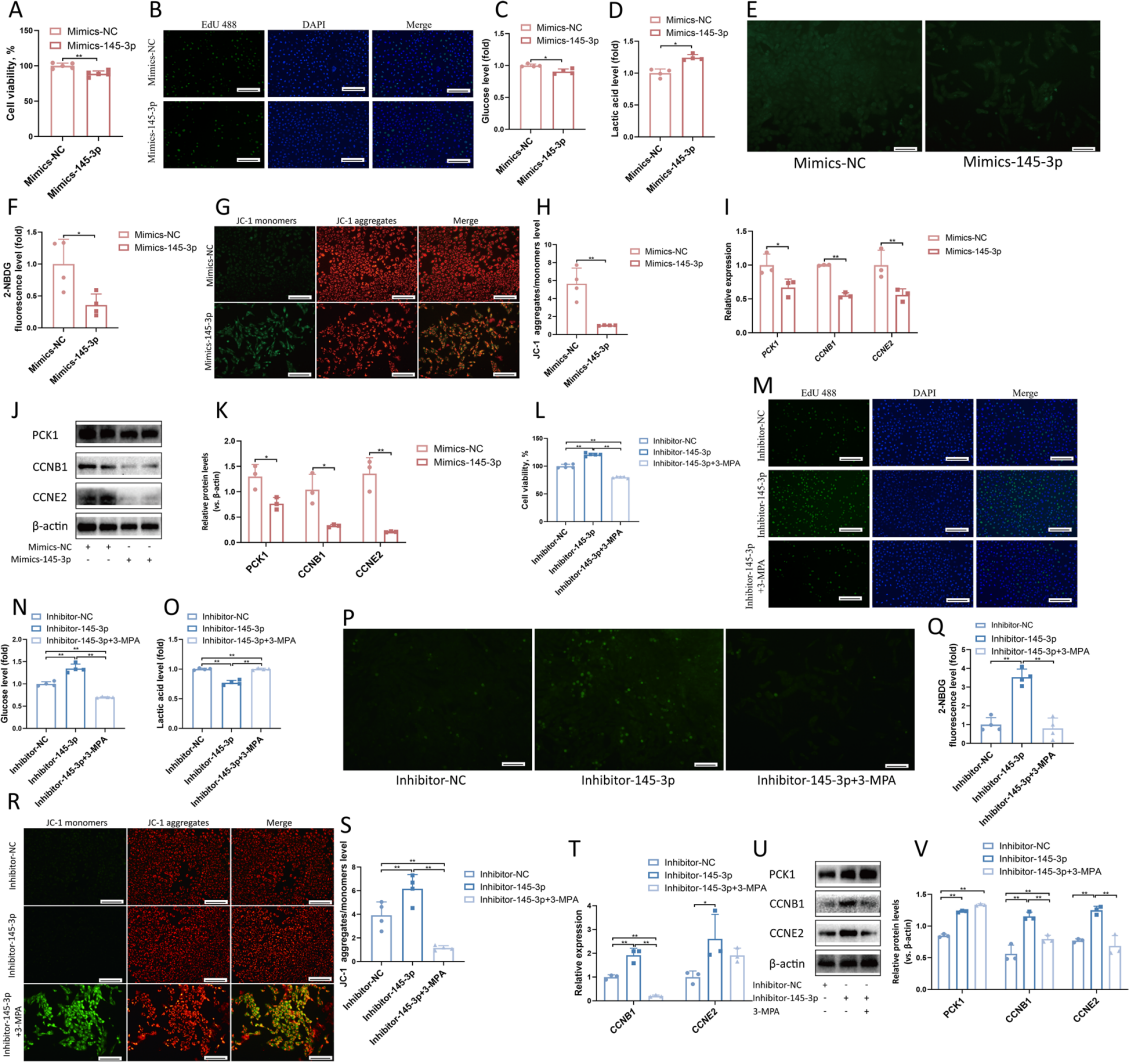
miR-145-3p regulates goat LC proliferation through the glyconeogenesis pathway. **A** Cell viability of LCs treated with Mimics-NC and Mimics-145-3p (*n* = 5). **B** LCs were treated with Mimics-NC and Mimics-145-3p, and EdU (green) was used to label the proliferating cells. The cell nuclei were counterstained with DAPI (*n* = 4). Scale bar: 100 μm. **C** Glucose level of LCs treated with Mimics-NC and Mimics-145-3p (*n* = 4). **D** Lactic acid level of LCs treated with Mimics-NC and Mimics-145-3p (*n* = 4). **E**–**F** Glucose uptake ability of LCs treated with Mimics-NC and Mimics-145-3p, assessed using immunofluorescence and quantitative analysis (*n *= 4). Scale bar: 100 μm. **G**–**H** Changes in mitochondrial membrane potential in LCs treated with Mimics-NC and Mimics-145-3p were analyzed by JC-1 staining and the fluorescence intensity of JC-1 aggregates/monomers was quantified (*n* = 4). Scale bar: 100 μm. **I** The relative mRNA expression levels of *PCK1*, *CCNB1* and *CCNE2* treated with Mimics-NC and Mimics-145-3p (*n* = 3). **J**–**K** The levels of PCK1, CCNB1, CCNE2 and β-actin were assessed via Western blotting. Relative protein levels were analysed via gray scanning (*n* = 3). **L** Cell viability of LCs treated with Inhibitor-145-3p and Inhibitor-145-3p+3-MPA (*n* = 5). **M** LCs were treated with Inhibitor-145-3p and Inhibitor-145-3p+3-MPA, and EdU (green) was used to label the proliferating cells. The cell nuclei were counterstained with DAPI (*n* = 4). Scale bar: 100 μm. **N** Glucose level of LCs treated with Inhibitor-145-3p and Inhibitor-145-3p+3-MPA (*n* = 4). **O** Lactic acid level of LCs treated with Inhibitor-145-3p and Inhibitor-145-3p+3-MPA (*n* = 4). **P**–**Q** Glucose uptake ability of LCs treated with Inhibitor-145-3p and Inhibitor-145-3p+3-MPA, assessed using immunofluorescence and quantitative analysis (*n* = 4). Scale bar: 100 μm. **R**–**S** Changes in mitochondrial membrane potential in LCs treated with Inhibitor-145-3p and Inhibitor-145-3p+3-MPA were analyzed by JC-1 staining and the fluorescence intensity of JC-1 aggregates/monomers was quantified (*n* = 4). Scale bar: 100 μm. **T** The relative mRNA expression levels of *CCNB1* and *CCNE2* treated with Inhibitor-145-3p and Inhibitor-145-3p+3-MPA (*n* = 3). **U**–**V** The levels of PCK1, CCNB1, CCNE2 and β-actin were assessed via Western blotting. Relative protein levels were analysed via gray scanning (*n* = 3). *P < 0.05, **P < 0.01

We further investigated the important role of miR-145-3p in LCs by increasing its expression using a specific mimic of miR-145-3p (hereinafter referred to as mimecs-miR-145-3p). CCK8 detection showed a significant decrease in cell viability after mimecs-miR-145-3p treatment (Fig. 6A), and EdU staining showed that the LC proliferation rate in the mimecs-miR-145-3p group was significantly slower than that in the modulate group (Fig. 6B). We explored the potential mechanism by which miR-145-3p promotes LC proliferation by measuring glucose levels in the mimecs-miR-145-3p group. As shown in Fig. 6C–D, glucose levels were reduced in the mimics-miR-145-3p group, while lactic acid levels increased. LCs in the mimics-miR-145-3p group exhibited reduced glucose uptake and lower mitochondrial membrane potential (Fig. 6E–H). Subsequently, we evaluated the mRNA levels of the cell proliferation markers *CCD1* and *CCNE2* and the gluconeogenesis gene *PCK1* using RT-qPCR. Our results indicated that miR-145-3p overexpression significantly reduced the mRNA expression level of these three genes (Fig. 6I), consistent with the Western blotting results (Fig. 6J–K). To further investigate the effect of miR-145-3p on the gluconeogenesis pathway of LCs via PCK1 in the regulation of goat LC proliferation, LCs were treated with 3-MPA. As shown in Fig. 6L–V, inhibition of PCK1 activity by 3-MPA treatment suppressed gluconeogenesis in LCs, reduced their proliferation, and inhibited glucose synthesis, leading to decreased glucose uptake and mitochondrial membrane potential, while increasing lactic acid levels. Additionally, the expression of the proliferation genes (*CCND1* and *CCNE2*) was significantly reduced. These results indicate that inhibition of miR-145-3p promotes *PCK1* expression, which in turn promotes LC gluconeogenesis and proliferation.

### PCK1 regulates goat LC proliferation through the gluconeogenesis pathway

**Fig. 7:**
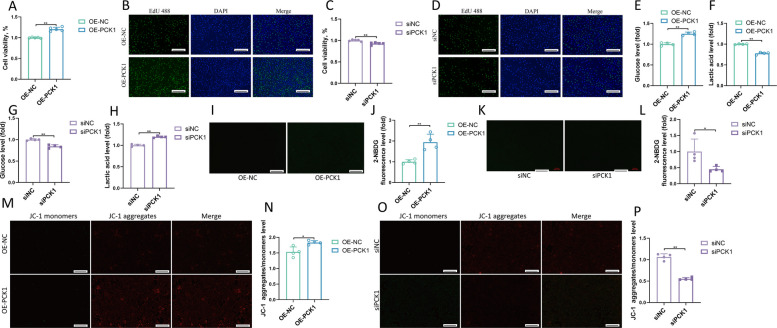
PCK1 promotes goat Leydig cell proliferation. **A** Cell viability of LCs treated with OE-NC and OE-PCK1 group (*n *= 5). **B** LCs were treated with OE-NC and OE-PCK1, and EdU (green) was used to label the proliferating cells. The cell nuclei were counterstained with DAPI (*n* = 4). Scale bar: 100 μm. **C** Cell viability of LCs treated with siNC and siPCK1 group (*n* = 5). **D** LCs were treated with siNC and siPCK1, and EdU (green) was used to label the proliferating cells (*n* = 4). **E** Glucose level of LCs treated with OE-NC and OE-PCK1 (*n *= 4). **F** Lactic acid level of LCs treated with OE-NC and OE-PCK1 (*n *= 4). **G** Glucose level of LCs treated with siNC and siPCK1 (*n* = 4). **H** Lactic acid level of LCs treated with siNC and siPCK1 (*n* = 4). **I**–**J** Glucose uptake ability of LCs treated with OE-NC and OE-PCK1, assessed using immunofluorescence and quantitative analysis (*n* = 4). Scale bar: 100 μm. **K**–**L** Glucose uptake ability of LCs treated with siNC and siPCK1, assessed using immunofluorescence and quantitative analysis (*n* = 4). Scale bar: 100 μm. **M**–**N** Changes in mitochondrial membrane potential in LCs treated with OE-NC and OE-PCK1 were analyzed by JC-1 staining and the fluorescence intensity of JC-1 aggregates/monomers was quantified (*n* = 4). Scale bar: 100 μm. **O**–**P** Changes in mitochondrial membrane potential in LCs treated with siNC and siPCK1 were analyzed by JC-1 staining and the fluorescence intensity of JC-1 aggregates/monomers was quantified (*n* = 4). Scale bar: 100 μm. *P < 0.05, **P < 0.01

**Fig. 8:**
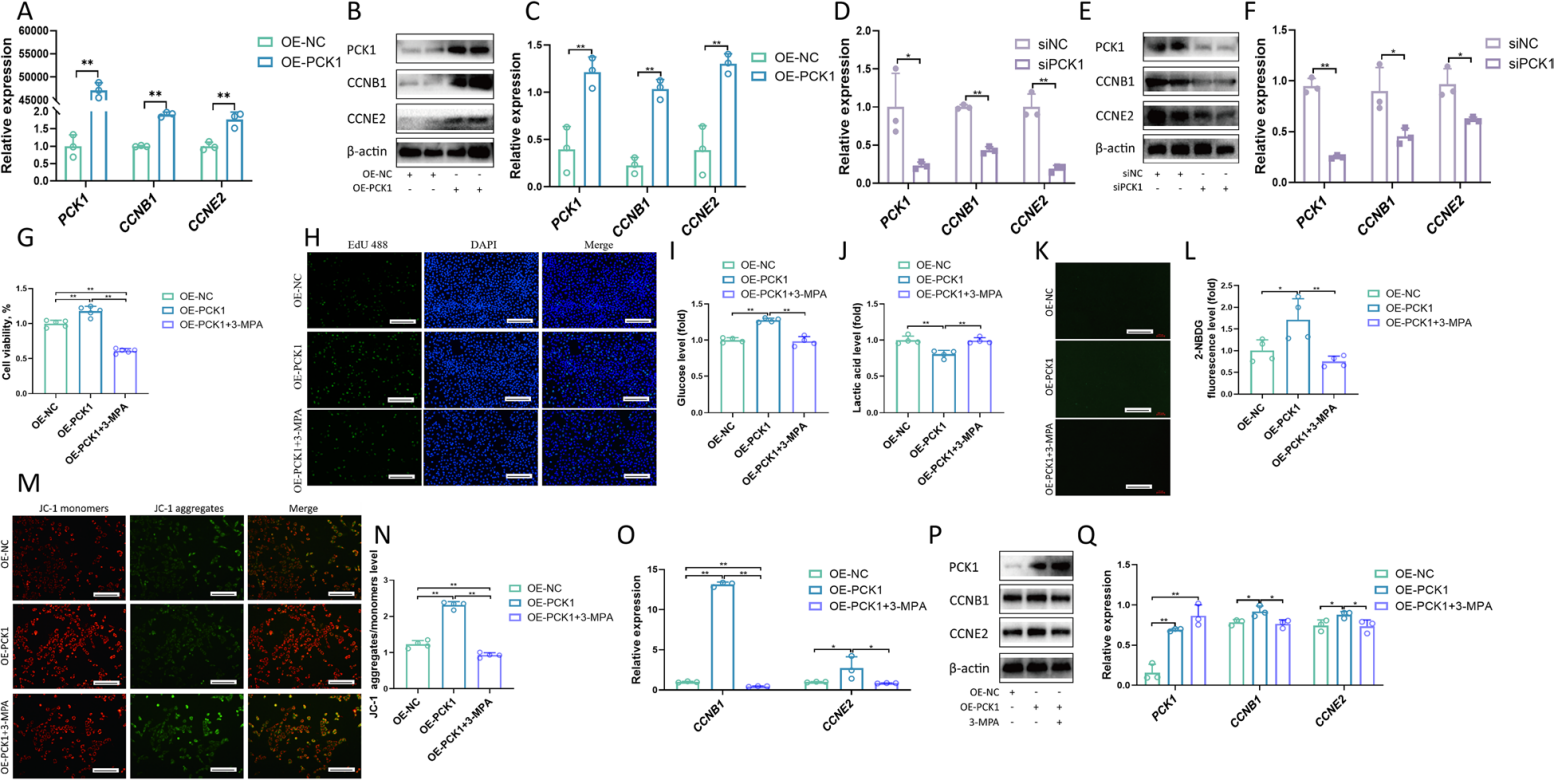
PCK1 regulates goat Leydig cell proliferation through the gluconeogenesis pathway. **A** The relative mRNA expression levels of *PCK1*, *CCNB1* and *CCNE2* treated with OE-NC and OE-PCK1 (*n *= 3). **B**–**C** The levels of PCK1, CCNB1, CCNE2 and β-actin were assessed via Western blotting. Relative protein levels were analysed via gray scanning (*n* = 3). **D** The relative mRNA expression levels of *PCK1*, *CCNB1* and *CCNE2* treated with siNC and siPCK1 (*n* = 3). **E**–**F** The levels of PCK1, CCNB1, CCNE2 and β-actin were assessed via Western blotting. Relative protein levels were analysed via gray scanning (*n* = 3). **G** Cell viability of LCs treated with OE-PCK1 and OE-PCK1+3-MPA group (*n* = 5). **H** LCs were treated with OE-PCK1 and OE-PCK1+3-MPA group, and EdU (green) was used to label the proliferating cells. The cell nuclei were counterstained with DAPI (*n* = 4). Scale bar: 100 μm. **I **Glucose level of LCs treated with OE-PCK1 and OE-PCK1+3-MPA (*n* = 4). **J** Lactic acid of LCs treated with OE-PCK1 and OE-PCK1+3-MPA (*n* = 4). **K–L** Glucose uptake ability of LCs treated with OE-PCK1 and OE-PCK1+3-MPA, assessed using immunofluorescence and quantitative analysis (*n* = 4). Scale bar: 100 μm. **M–N **Changes in mitochondrial membrane potential in LCs treated with OE-PCK1 and OE-PCK1+3-MPA were analyzed by JC-1 staining and the fluorescence intensity of JC-1 aggregates/monomers was quantified (*n* = 4). Scale bar: 100 μm. **O** The relative mRNA expression levels of *CCNB1* and *CCNE2* treated with OE-PCK1 and OE-PCK1+3-MPA (*n* = 3). **P**–**Q** The levels of PCK1, CCNB1, CCNE2 and β-actin were assessed via Western blotting. Relative protein levels were analysed via gray scanning (*n* = 3). *P < 0.05, **P < 0.01

To investigate the regulatory effects of PCK1 on goat LCs, we overexpressed (OE-PCK1) and inhibited (siPCK1) PCK1 expression in LCs. The CCK8 and EdU assays showed that the OE-PCK1 group had a significantly increased cell proliferation rate (Fig. 7A–B) whereas the siPCK1 group had the opposite effect (Fig. 7C–D). We explored the potential mechanism by which *PCK1* promotes LC proliferation by measuring glucose levels in the OE-PCK1 and siPCK1 groups. As shown in Fig. 7E–H, glucose levels increased and lactic acid levels decreased in the OE-PCK1 group, whereas glucose levels decreased and lactic acid levels increased in the siPCK1 group. Analysis of glucose uptake levels by LCs showed that the OE-PCK1 group had increased glucose uptake (Fig. 7I–J) whereas the siPCK1 group had decreased glucose uptake (Fig. 7K–L). Analysis of mitochondrial membrane potential levels showed that the OE-PCK1 group had increased mitochondrial membrane potential levels whereas the siPCK1 group had decreased mitochondrial membrane potential levels (Fig. 7M–P). Furthermore, RT-PCR revealed that the OE-PCK1 group had significantly increased mRNA levels of the gluconeogenic genes *PCK1*, *CCNB1*, and *CCNE2* in LCs (Fig. 8A), consistent with the Western blotting results (Fig. 8B–C). In contrast, the siPCK1 group showed significantly inhibited mRNA levels of *PCK1*, *CCNB1*, and *CCNE2* in LCs (Fig. 8D), consistent with the Western blotting results (Fig. 8E–F). We further investigated how METTL3 regulates goat LC proliferation through the PCK1-regulated gluconeogenesis pathway in LCs via treatment with the PCK1 inhibitor 3-MPA. Inhibition of *PCK1* activity by 3-MPA increased the inhibition of gluconeogenesis in LCs and decreased their proliferation. It also inhibited glucose synthesis and increased lactic acid levels, resulting in reduced glucose uptake and mitochondrial activity, as well as significant downregulation of proliferation-related genes and their corresponding proteins (Fig. 8G–Q). These results indicate that the increased expression of *PCK1* promotes cell proliferation by promoting gluconeogenesis in LCs.

## Discussion

Normal testis development is a key factor determining the fertility of rams. Therefore, studying the regulatory mechanisms of ram testicular development is important for improving the semen quality of male animals, preventing or treating reproductive disorders caused by abnormal testicular development, and improving ram fertility. As one of the earliest germ cells, LCs play an indispensable role in the reproductive process of testicular development [20]. In mammals, they promote testicular development and androgen secretion and may improve the reproductive ability by promoting LC proliferation and androgen secretion [20, 21]. However, the molecular mechanisms that regulate LC proliferation is not fully understood and require further investigation.

RNA m^6^A modification has been shown to be involved in many important physiological and pathological processes in the reproductive system [22]. m^6^A modifies the 3' untranslated region (UTR) of longer mRNAs that may be degraded during spermatogenesis, and shortening the 3' UTR as a whole by selectively degrading longer 3' UTR transcripts is essential to improve translation efficiency and rapid turnover in spermatogenesis [23]. The demethylases FTO and *ALKBH5* are highly expressed in spermatocytes and round spermatids of the testes. *ALKBH5* knockout in mice leads to impaired male fertility, characterized by impaired spermatogenesis, increased apoptosis of meiotic cells, and altered meiotic metaphase spermatocytes [24]. *ALKBH5* regulates mRNA splicing and stability. *ALKBH5*-mediated m^6^A modification is indispensable for proper mRNA splicing and the generation of longer 3' UTR transcripts [23]. During the later stages of spermatogenesis, m^6^A modifications are extensively enriched in genes essential for sperm production, suggesting that m^6^A-dependent RNA translation represents a regulatory mechanism modulating late spermatogenesis [6]. m^6^A levels are significantly elevated in patients with asthenozoospermia. Mechanistic investigations have demonstrated that *METTL3* plays a pivotal role in augmenting m^6^A enrichment in spermatozoal RNA [25]. In addition to its function in spermatogenic cells, m^6^A participates in spermatogenesis by modulating Sertoli cell activity. Conditional knockout of *WTAP*, specifically in Sertoli cells, induces male infertility accompanied by severe dysregulation of alternative splicing in spermatogonial stem cells, establishing the indispensable role of *WTAP* in maintaining the spermatogonial stem cell niche [26]. Exogenous supplementation with vitamin C in vitro reduces m^6^A levels in immature porcine Sertoli cells, thereby enhancing reproductive function [27]. Furthermore, m^6^A modifications regulate testosterone synthesis in LCs via the autophagic pathway [28]. In the present study, *METTL3* expression progressively decreased in caprine LCs during testicular development.

miRNAs are noncoding RNA molecules composed of 19–25 nucleotides encoded by the eukaryotic genome. In the nucleus, RNA polymerase II transcribes the encoded gene into a longer transcript called pri-miRNA, which then becomes a pre-miRNA under the action of the RNA-binding protein DGCR8. Subsequently, the pre-miRNA enters the cytoplasm and is processed into a mature miRNA by Dicer [29]. Studies have shown that miRNAs play crucial roles in regulating testicular development. Knockout of the key miRNA maturation gene *DICER1* in germ cells disrupts spermatogenesis, resulting in infertility [29]. The deletion of *DICER1* in supporting cells results in oxidative damage in supporting and germ cells, ultimately inducing cell apoptosis [29]. When miRNA-10a is overexpressed in mouse germ cells, testicular weight decreases by 50%, germ cell meiosis becomes abnormal, and infertility ultimately occurs [30]. In addition, miR-10a overexpression can promote the differentiation of undifferentiated spermatogonia and reduce the number of spermatogonia entering meiosis [30]. Overexpression of miR-130 in supporting cells inhibits the expression of androgen receptors, ultimately leading to germ cell apoptosis [31]. In this study, we found that mature miRNAs decreased as the goat testes developed. m^6^A is not only the most abundant RNA methylation modification in mRNAs, but is also present in noncoding RNAs (lncRNAs, pri-miRNA). m^6^A modification plays an important role in pri-miRNA processing [2]. *METTL3* is a key methyltransferase in m^6^A modification, which not only affects the stability of mRNAs but also affects the synthesis of miRNAs [2]. Our previous study showed that METTL3 positively regulates pri-miR-21 synthesis in an m^6^A-dependent manner by interacting with DGCR8, thereby regulating follicular development and the occurrence of follicular cystic diseases [32, 33]. miR-145-3p plays an important role in regulating cell proliferation and inflammation. In prostate cancer cells, a decrease in its expression promotes the expression of proliferation-related genes *MELK*, *NCAPG*, *BUB1*, and *CDK1*, thereby promoting cell proliferation [34]. In rats with traumatic brain injury, miR-145-3p regulates the balance between Th1 and Treg cells through the NFATc2/NF-κB axis, thereby reducing inflammation [35]. In mice, miR-145-5p overexpression in glial cells can inhibit the inflammatory response and maintain M2 macrophage polarization by targeting TLR4/NF-κB signaling, thereby promoting the tissue repair of spinal cord injury in mice [36]. In the present study, the reduction of *METTL3* in LCs led to a decrease in the m^6^A modification level of pri-miR-145, which reduced the recognition and binding of DGCR8 to pri-miR-145, resulting in a decrease in mature miR-145-3p synthesis and ultimately leading to an increase in PCK1 expression and promotion of LC proliferation.

In the present study, we identified *PCK1* as a direct target of miR-145-3p in goat LCs, indicating that it plays a critical role in testicular development. PCK1 is the first rate-limiting enzyme in the gluconeogenesis pathway, which is the reverse of glycolysis. It converts non-carbohydrate carbon sources, such as lactate, amino acids, and triglycerides, into glucose to maintain glucose homeostasis and meet the body's energy needs. Many studies have shown that the key gene *PCK1* in gluconeogenesis plays a crucial role in disease occurrence and treatment. In patients with fatty liver, the mRNA level of *PCK1* is reduced. Mice lacking *PCK1* in the liver can develop hepatitis and fibrosis, resulting in a fatty liver phenotype and liver damage. Studies have reported that PCK1 causes liver metabolic disorders via the PI3K/AKT/PDGF axis, ultimately leading to fatty liver disease [37]. In addition, studies have shown that METTL3 regulates liver gluconeogenesis by influencing the m^6^A modification level of PCK1. PCK1 has been shown to be a potential therapeutic target for alleviating liver ischemia–reperfusion injury [38]. It is crucial for mitochondrial function in diabetes nephropathy. The absence of *PCK1* in renal tubular epithelial cells causes mitochondrial ribosome defects and a reduction in the mitochondrial membrane potential, which ultimately leads to mitochondrial dysfunction. *PCK1* overexpression inhibits renal fibrosis by protecting the mitochondrial ribosome [39]. Taken together, these studies indicate that PCK1 affects body development by influencing the gluconeogenic pathway and mitochondrial function. In the present study, we found that *PCK1* expression gradually increased during testicular development. Additional mechanistic studies suggested that the METTL3-mediated reduction in m^6^A modification in LCs affects the recognition and binding of DGCR8 to pri-miR-145, leading to a decrease in miR-145-3p synthesis. Reduction in miR-145-3p expression promotes the expression of *PCK1*, which promotes gluconeogenesis in LCs that leads to increased glucose synthesis and mitochondrial activity, further upregulating the proliferation genes *CCNB1* and *CCNE2* and ultimately promoting LC proliferation. Notably, this study is the first to reveal a new signal transduction pathway during testicular development, METTL3/miR-145-3p/PCK1, which not only promotes LC gluconeogenesis and proliferation, but also enriches the testicular development network from the perspective of m^6^A modification.

This study is the first to reveal insight into a new signalling transduction pathway in testicular development, “METTL3/miR-145-3p/PCK1”. It elucidated the relationship between gluconeogenesis and proliferation of LCs and showed the network of testicular development from the perspective of m^6^A modification. However, PCK1 is just one of the key enzymes in gluconeogenesis, and the precise signalling mechanism of gluconeogenesis in LCs remains unknown. Further research is needed to validate its function in vivo. Future studies should focus on elucidating the key role of the METTL3/miR-145-3p/PCK1 network in vivo and the effect of other key enzymes in gluconeogenesis on the development ofis.

## Conclusion

**Fig. 9:**
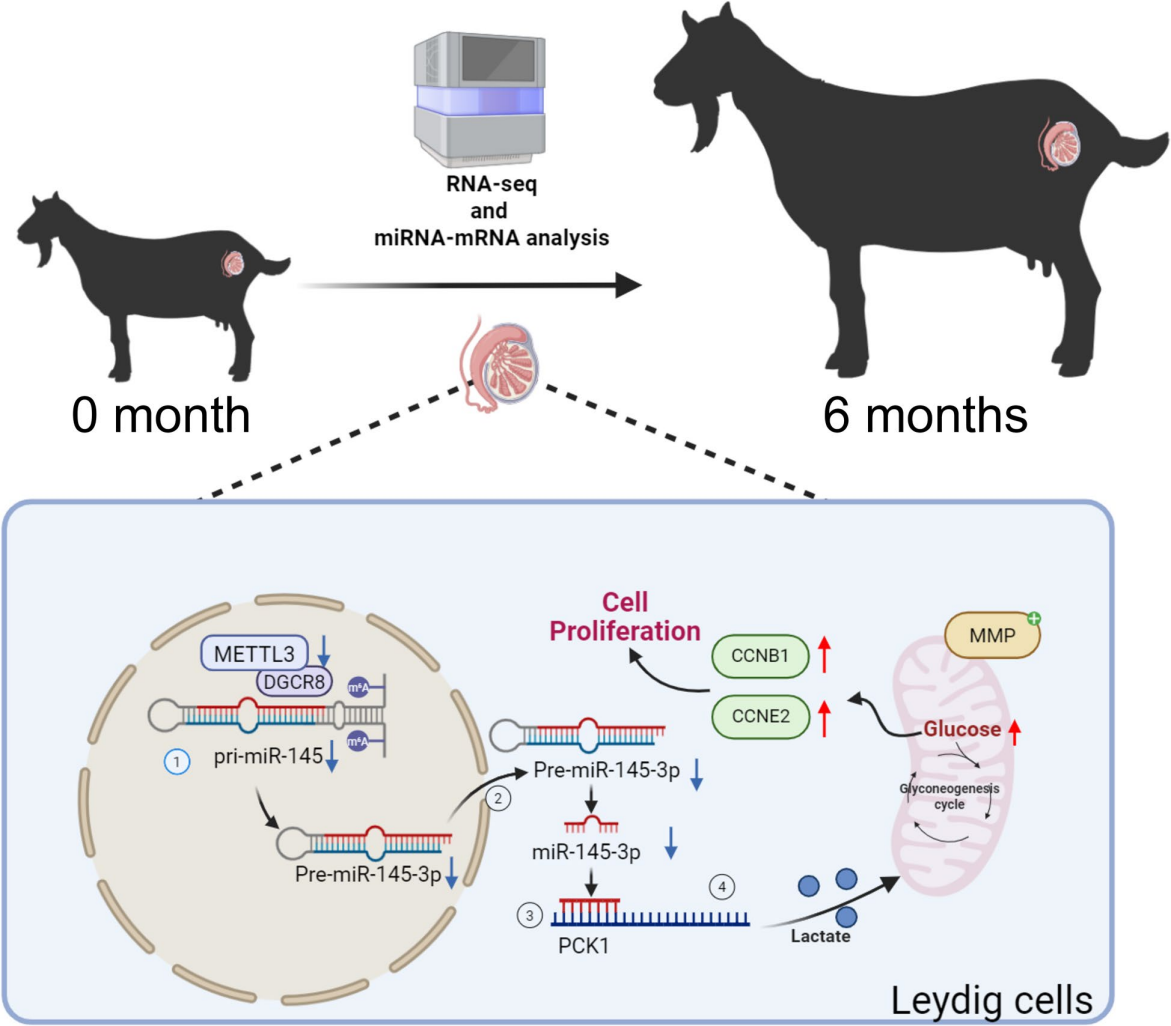
As the testis develops, a model diagram of *METTL3* gene reducing the promotion of Leydig cells proliferation

Our findings indicate that as the testes develop, the levels of *METTL3* and miR-145-3p decrease. Further investigation confirmed the importance of the METTL3/miR-145-3p/PCK1 gluconeogenesis pathway in the regulation of LC function and physiological activity in male goat testes (Fig. 9). In addition, we identified *PCK1* as a novel target of miR-145-3p and established a signaling pathway for modulating LC proliferation through gluconeogenesis. Importantly, our results emphasize the potential role of METTL3/miR-145-3p/PCK1 as an epigenetic modification pathway for testicular development in goats. Targeting this pathway may be a promising approach to regulate LC proliferation, promote testicular development, and improve male reproductive performance.

## Supplementary Information

Additional file 1: Table S1. siRNAs used in this study. Table S2. Primers used in qRT-PCR assays. Table S3. Antibody information. Fig. S1. Immunofluorescence identification of testicular Leydig cells. Fig. S2. Upregulated differential genes KEGG pathway enrichment analysis. Fig. S3. Expression level of METTL3 in goat testicular Leydig cells. Fig. S4. miR-145-3p and PCK1 double luciferin analysis.

## Data Availability

The datasets generated and/or analyzed during the current study are available from the corresponding author upon reasonable request.

## Abbreviations

3-MPA: 3-Mercaptopicolinic acid
AKT: Protein kinase B
*ALKBH5*: AlkB homolog 5
AMPK: AMP-activated protein kinase
ATP: Adenosine triphosphate
CCK8: Cell Counting Kit-8
*CCNB1*: Cyclin B1
*CCNE2*: Cyclin E2
DEPC: Diethylpyrocarbonate
DGCR8: DiGeorge syndrome critical region 8
DNMT3b: DNA methyltransferase 3 beta
EdU: 5-Ethynyl-2'-deoxyuridine
FTO: Fat mass and obesity-associated protein
GATA4: GATA binding protein 4
HE: Hematoxylin and eosin
ID4: Inhibitor of DNA binding 4
*IGF1*: Insulin-like growth factor 1
*INSL3*: Insulin-like 3
KEGG: Kyoto Encyclopedia of Genes and Genomes
LCs: Leydig cells
lncRNAs: Long noncoding RNAs
m^6^A: N^6^-methyladenosine
MeRIP: Methylated RNA immunoprecipitation
*METTL3*: Methyltransferase like 3
*METTL14*: Methyltransferase like 14
mRNAs: Messenger RNAs
NF-κB: Nuclear factor kappa-light-chain-enhancer of activated B cells
*PCK1*: Phosphoenolpyruvate carboxykinase 1
PDGF: Platelet-derived growth factor
PI3k: Phosphoinositide 3-kinase
*PLZF*: Promyelocytic leukemia zinc finger protein
*TLR4*: Toll-like receptor 4
*WTAP*: Wilms tumor 1-associating protein
