# Supplementary material for: METTL3 regulates Leydig cell proliferation via miR-145-PCK1 mediated gluconeogenesis in goats
Source: J Anim Sci Biotechnol. 2026 Jan 17;17:10. doi: 10.1186/s40104-025-01307-5 (PMC12811904; doi:10.1186/s40104-025-01307-5)

M6A methyltransferase METTL3-mediated regulation of the glyconeogenic enzyme PCK1 controls the proliferation of Leydig cells in goats

Wen Tang^1,2,3^, Maosheng Cao^1,2,3^*, Fengxin Qiao^1,2,3^, Jinhong Luo^1,2,3^, Yonghong Ju^1,2,3^, Xiaodong Wang^1,2,3^, Pengchen An^1,2,3^, Wei Sun^4^, Xiang Chen^1,2,3^*

1Key Laboratory of Animal Genetics, Breeding and Reproduction in The Plateau Mountainous Region, Ministry of Education, Guizhou University, Guiyang 550025, China

^2^Key Laboratory of Animal Genetics, Breeding and Reproduction, Guiyang 550025, China

^3^College of Animal Science, Guizhou University, Guiyang 550025, China

^4^College of Animal Science and Technology, Yangzhou University, Yangzhou 225009, China

^*^Corresponding authors:

Maosheng Cao, Email: mscao@gzu.edu.cn, Phone: 0851-88298109

Mailing address: College of Animal Science, Guizhou University, Guiyang 550025, China

Xiang Chen, Email: xchen2@gzu.edu.cn, Phone: 0851-88298109

Mailing address: College of Animal Science, Guizhou University, Guiyang 550025, China

**Supplementary Materials**

**Supplementary Tables**

**Table S1.** siRNAs used in this study.

| **siRNAs** | **Sense (5'-3')** |
| --- | --- |
| Negative Control (goat) siRNA | UUCUCCGAACGUGUCACGU tt  ACGUGACACGUUCGGAGAA tt |
| *METTL3* (goat) siRNA-1 | GACCCUGAAUUAGAGAAGATT  UCUUCUCUAAUUCAGGGUCTT |
| *METTL3* (goat) siRNA-2 | CAGAUGGUAUCAUCUCUAATT  UUAGAGAUGAUACCAUCUGTT |
| *PCK1* (goat) siRNA-1 | GGAUGUGGCCAGAAUUGAATT  UUCAAUUCUGGCCACAUCCTT |
| *PCK1* (goat) siRNA-2 | GCCGAGAAAUCAUCUCCUUTT  AAGGAGAUGAUUUCUCGGCTT |
| inhibitor-Negative Control (goat) siRNA | UCUACUCUUUCUAGGAGGUUGUGA |
| inhibitor-miR-145-3p (goat) | AAGAACAGUAUUUCCAGGAAU |
| Mimics-Negative Control (goat) siRNA | UCACAACCUCCUAGAAAGAGUAGA |
| Mimics-miR-145-3p (goat) | AUUCCUGGAAAUACUGUUCUU  AAGAACAGUAUUUCCAGGAAU |

**Table S2.** Primers used in qRT-PCR assays.

| Gene | **Forward(5’-3’)** | **Reverse(5’-3’)** | **The annealing temperature (°C)** | **amplicon length(bp)** | **primer efficiency (%)** |
| --- | --- | --- | --- | --- | --- |
| *β-actin* (goat) | GGCATTCACGAAACTACCTTC | ATCTCTTTCTGCATCCTGTCTG | 60 | 134 | 99 |
| *METTL3* (goat) | ATGCTGTGTCTATCCGTCTTG | ATGTGCATCGTCTTGTAGGAG | 60 | 137 | 99 |
| *PCK1* (goat) | AGGGAGTTCGTGGAGAGTAG | TCTTCAGCCTCTTGATCACAC | 60 | 130 | 98 |
| *CCNB1* (goat) | CACCTACACCAAGTTTCAAATCAG | GTAGCTCAACATCAACCTCTCC | 60 | 134 | 98 |
| *CCNE2* (goat) | ACGCAGTAGCCGTTTACAAG | TGATGTTTCTTGGTGACCTCC | 60 | 144 | 98 |
| *U6* (goat) | CTCAGAATCACCCAATGC | ATGTTCATCCAGTTGTCAC | 60 | 85 | 99 |
| miR-145-3p (goat) | AGCTGGGATTCCTGGAAATACT | GCAGGGTCCGAGGTATTC | 60 | 86 | 99 |
| pri-miR-145 (goat) | TGGGGGCAACTTGGGTGGGAAG | GGATTCCTGGAAATACTGTTCTTG | 60 | 54 | 99 |

**Table S3.** Antibody information.

| Protein name | Manufacture (catalog number) | Applications (working dilution) |
| --- | --- | --- |
| METTL3 | Proteintech (15073-1-AP) | WB (1:1, 000), IHC (1:200), IP (1:50) |
| PCK1 | Proteintech (16754-1-AP) | WB (1:1, 000) |
| DGCR8 | Proteintech (60084-1-Ig) | WB (1:1, 000) |
| CCNB1 | Beyotime Biotechnology (AF6627) | WB (1:1, 000) |
| CCNE2 | Proteintech(11935-1-AP) | WB (1:1, 000) |
| β-actin | Proteintech (20536-1-AP) | WB (1:8 000) |
| Goat Anti-Mouse IgG (H+L) HRP | Proteintech (SA00001-1) | WB (1:10 000) |
| Goat Anti-Rabbit IgG (H+L) HRP | Proteintech (SA00001-2) | WB (1:10 000) |

**Supplementary Figure**


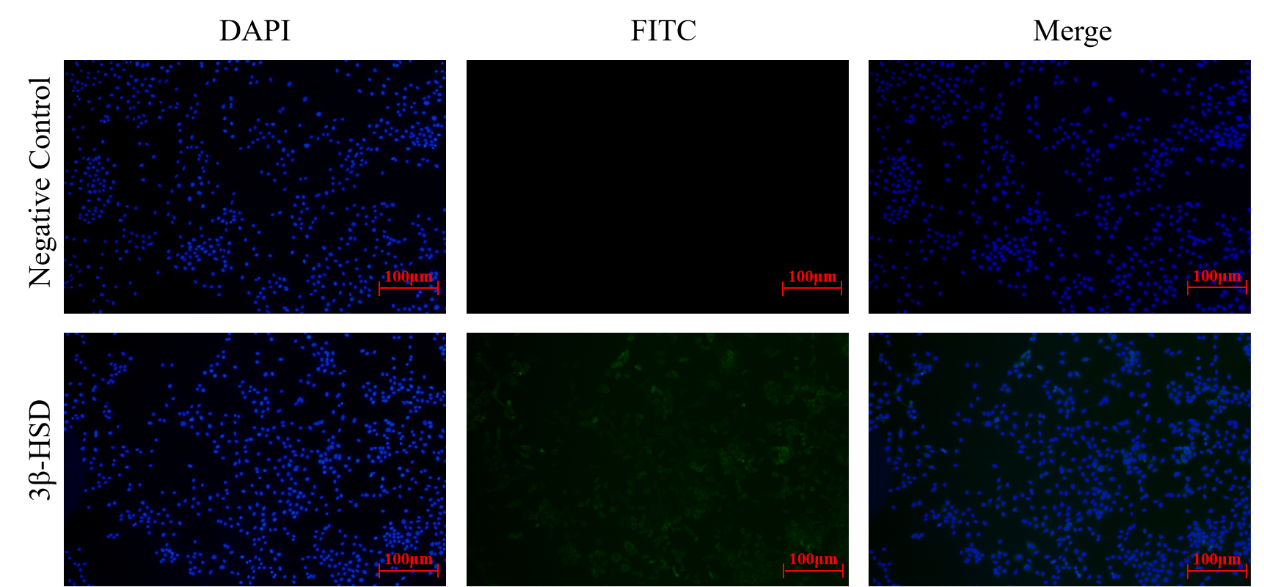


**Fig. S1 Immunofluorescence identification of testicular leydig cells.**


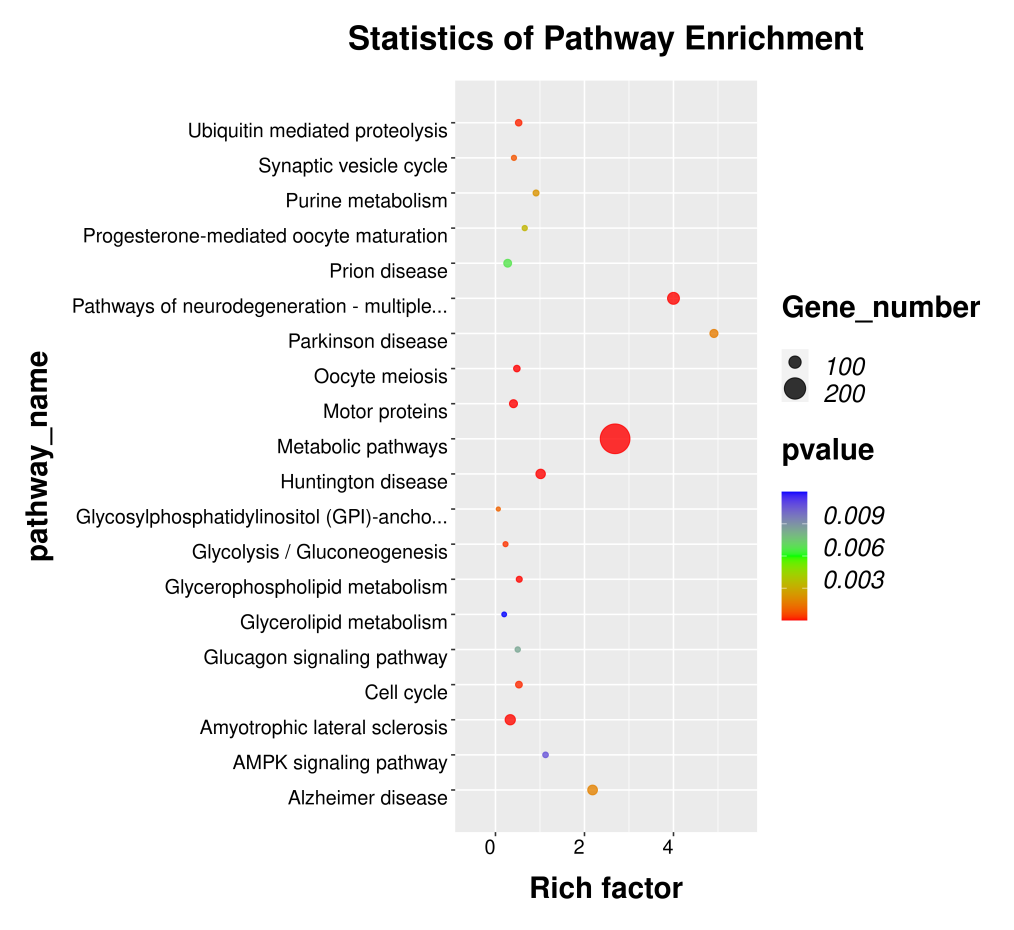


**Fig. S2 Upregulated differential genes KEGG pathway enrichment analysis.**


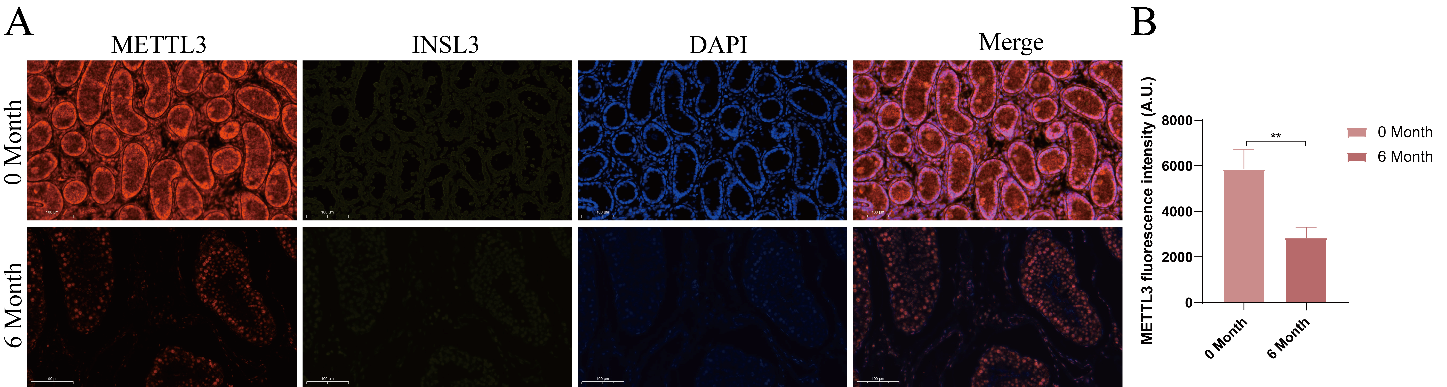


**Fig. S3 Expression level of METTL3 in goat testicular Leydig cells.**

**
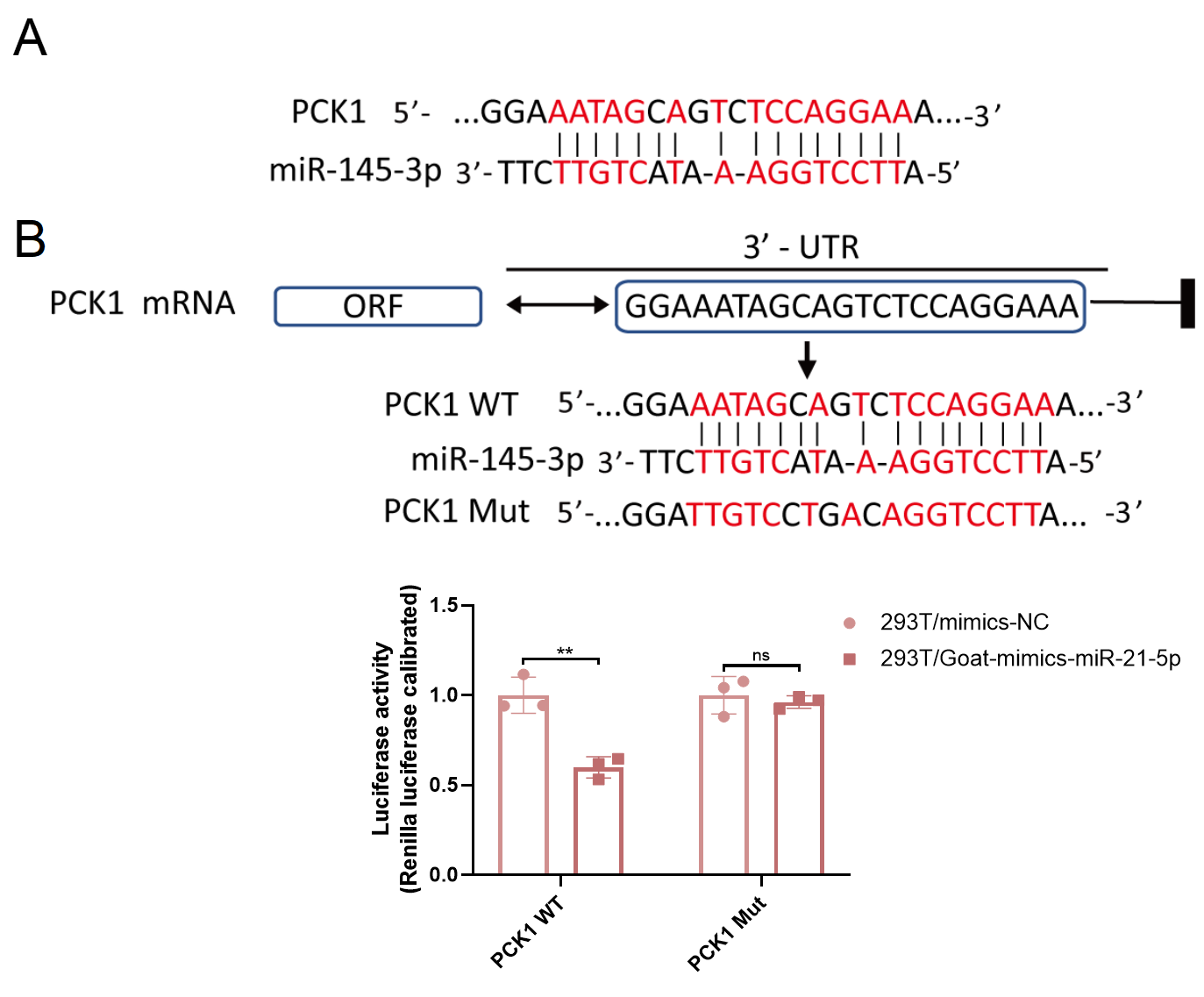
**

**Fig. S4 miR-145-3p and *PCK1* double luciferin analysis.**

**Original Western blot images corresponding to figures in the main text.**

Figure.2 J-METTL3


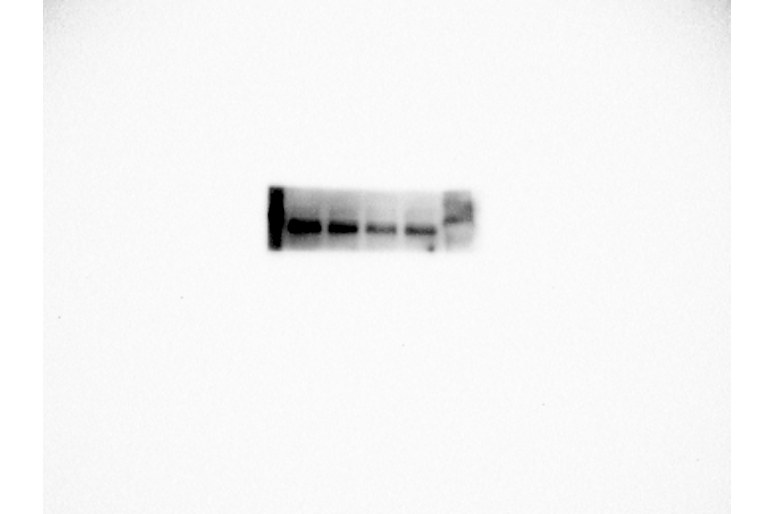


Figure.2 J-PCK1


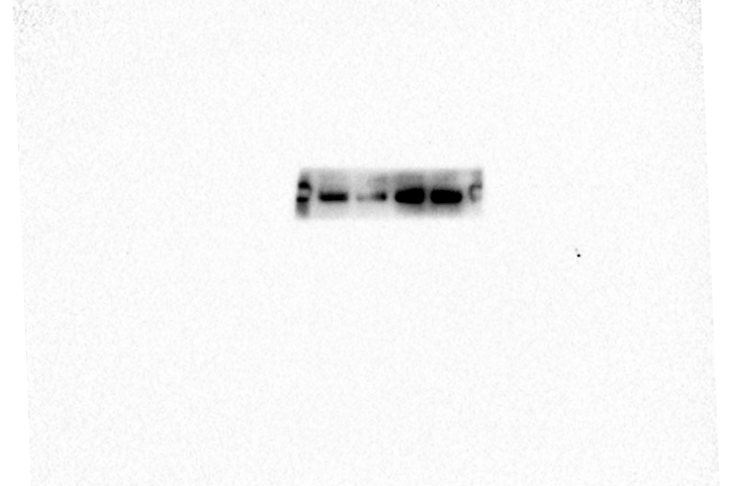


Figure.2 J-CCNB1


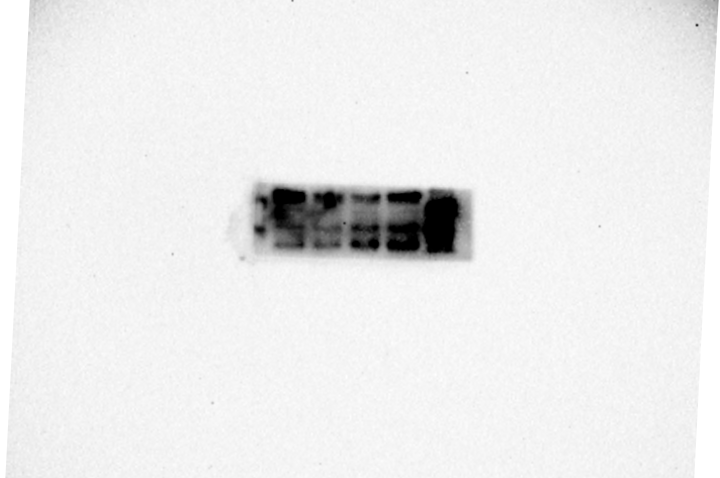


Figure.2 J-CCNE2


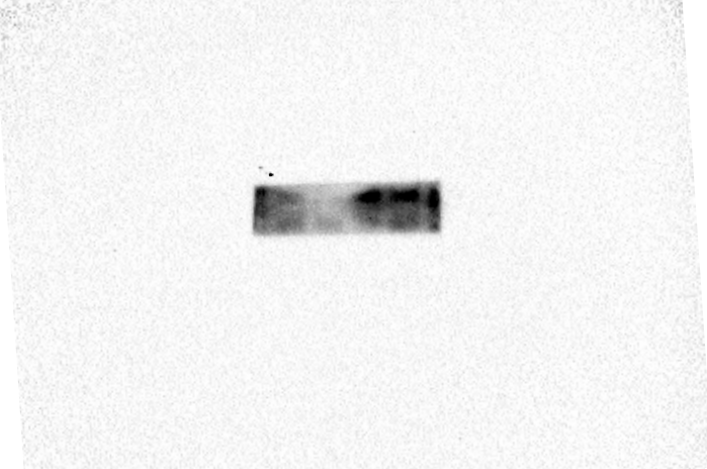


Figure.2 J-β-actin


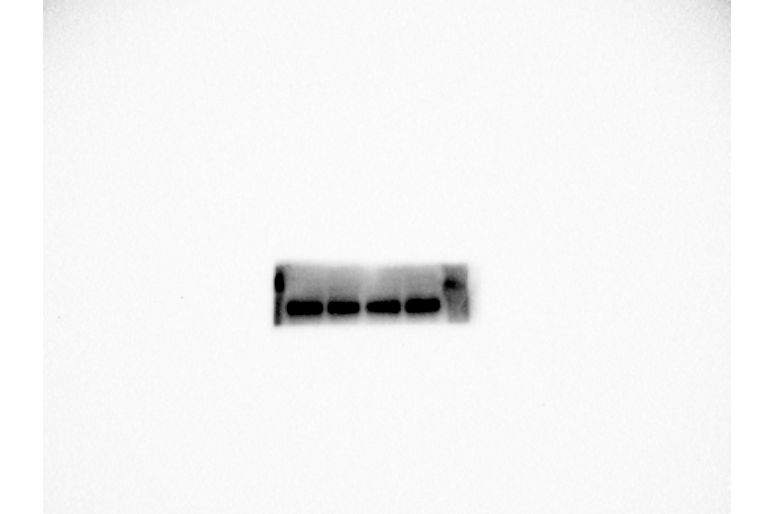


Figure.3 I-METTL3


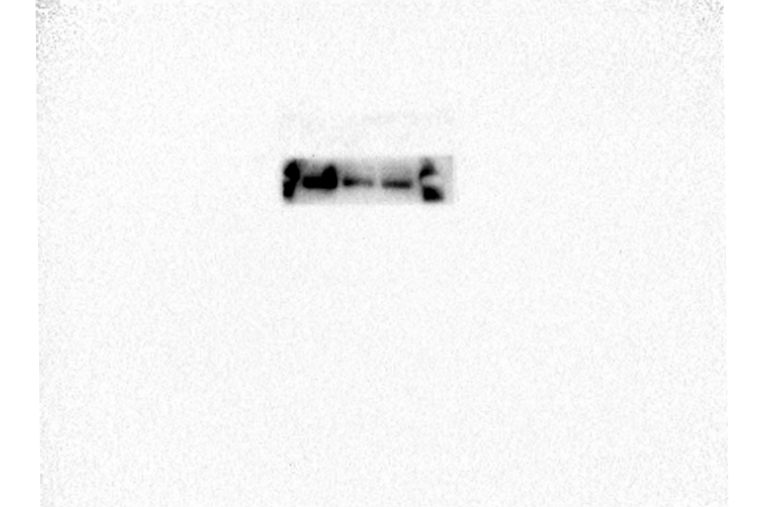


Figure.3 I-PCK1


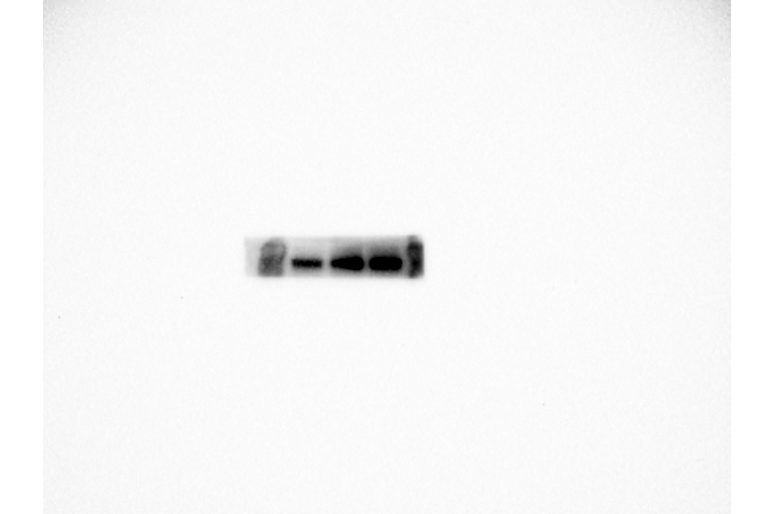


Figure.3 I-CCNB1


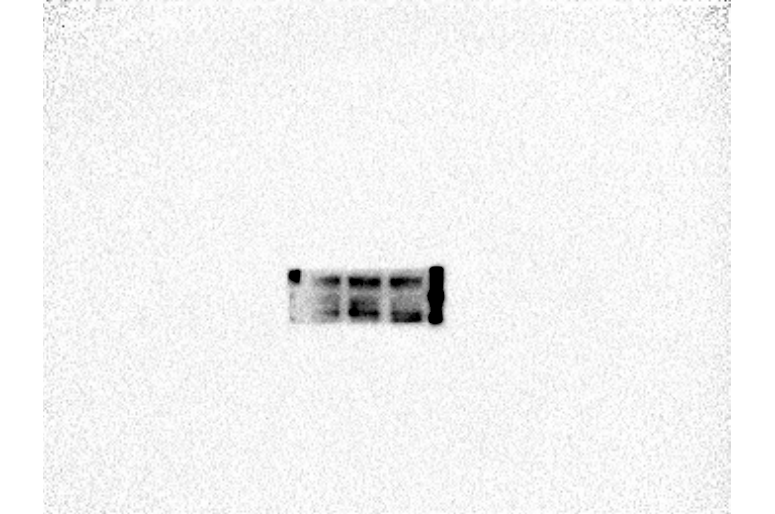


Figure.3 I-CCNE2


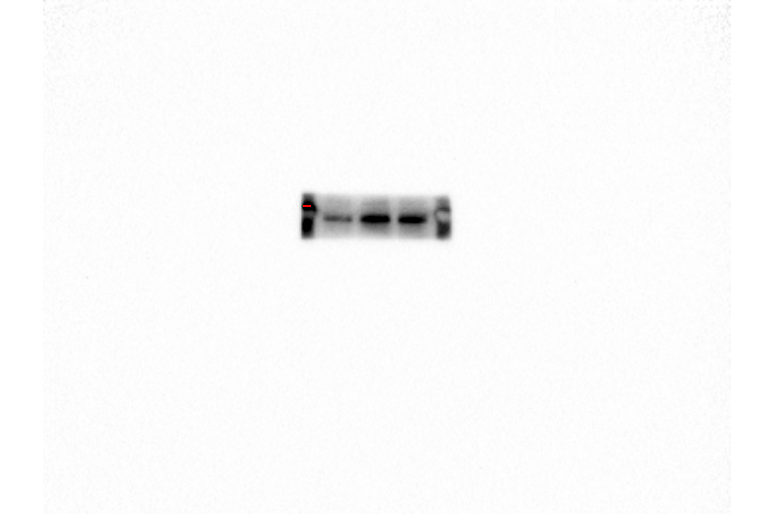


Figure.3 I-β-actin


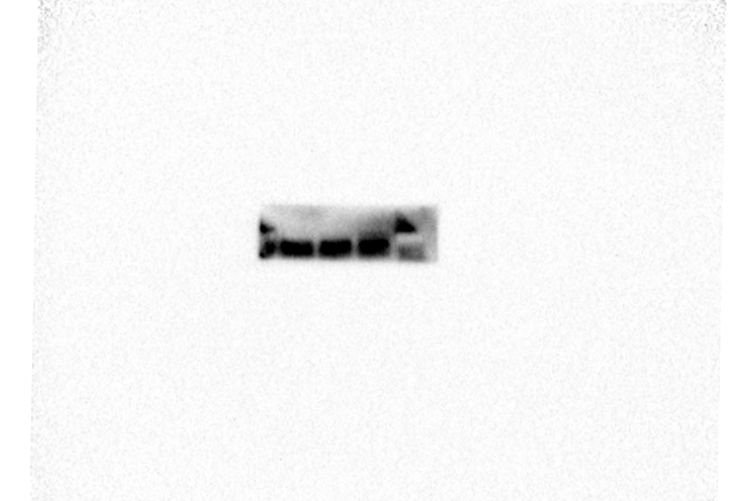


Figure.5 A-DGCR8


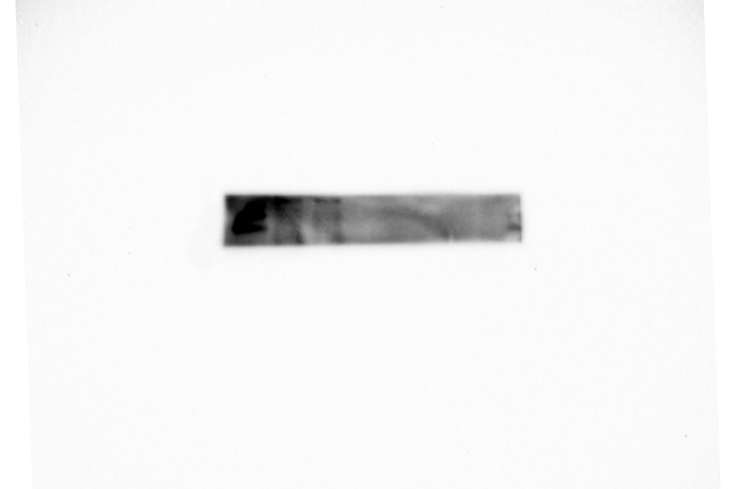


Figure.5A-METTL3
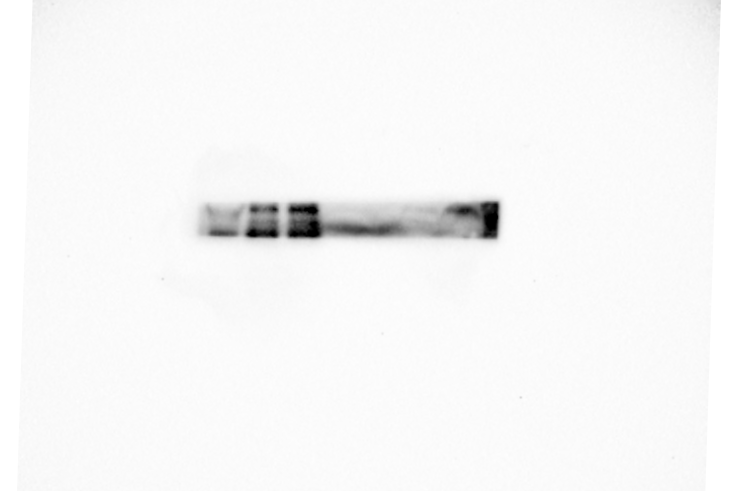


Figure.5 A-β-actin


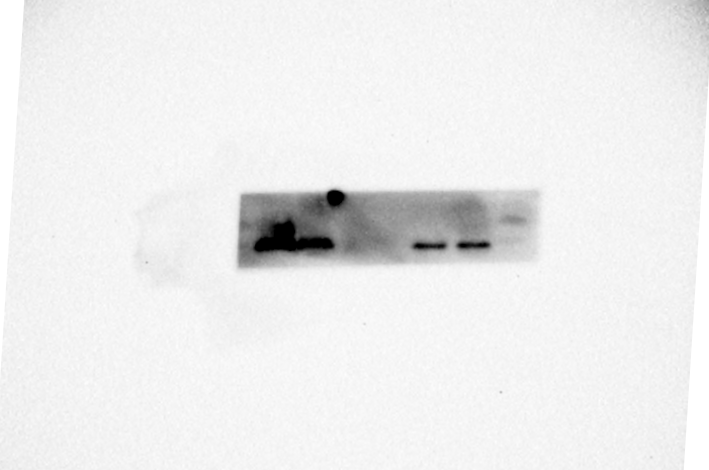


Figure.5 N-PCK1


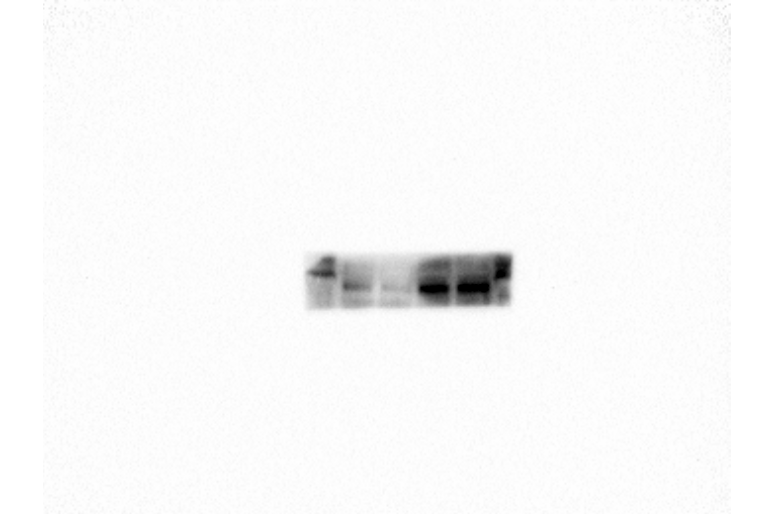


Figure.5 N-CCNB1


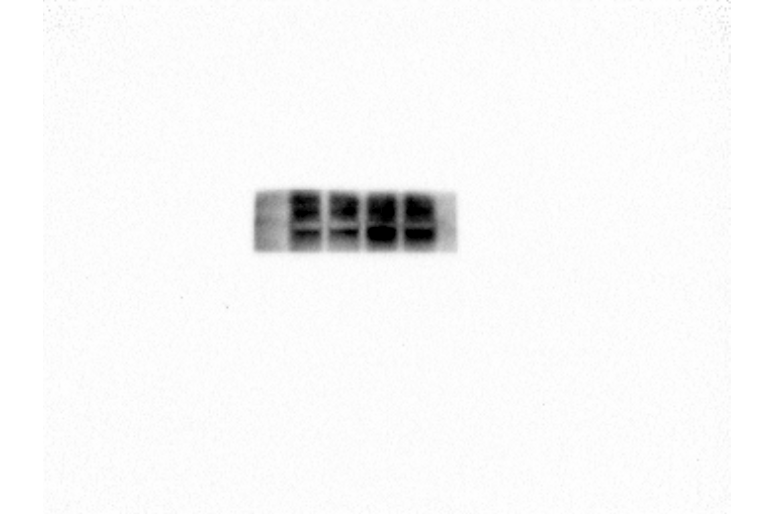


Figure.5 N-CCNE2


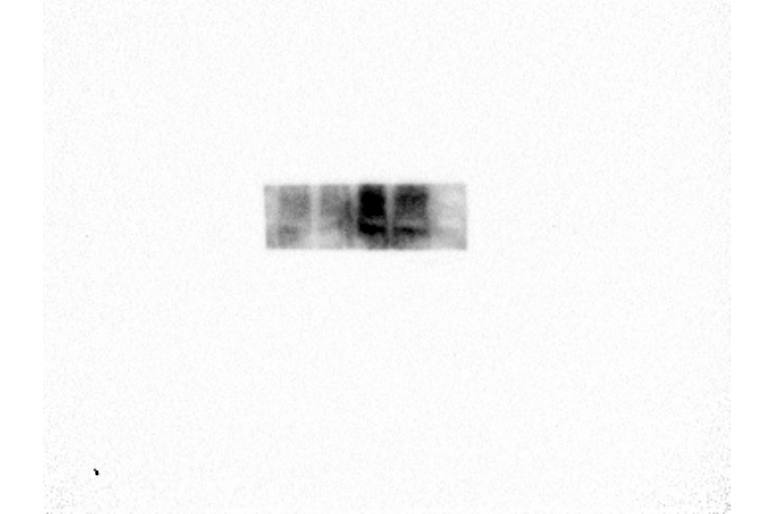


Figure.5 N-β-actin


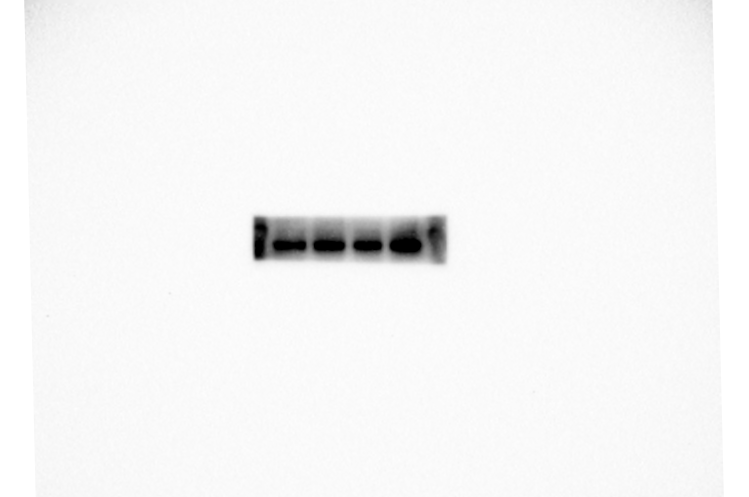


Figure.6 J-PCK1

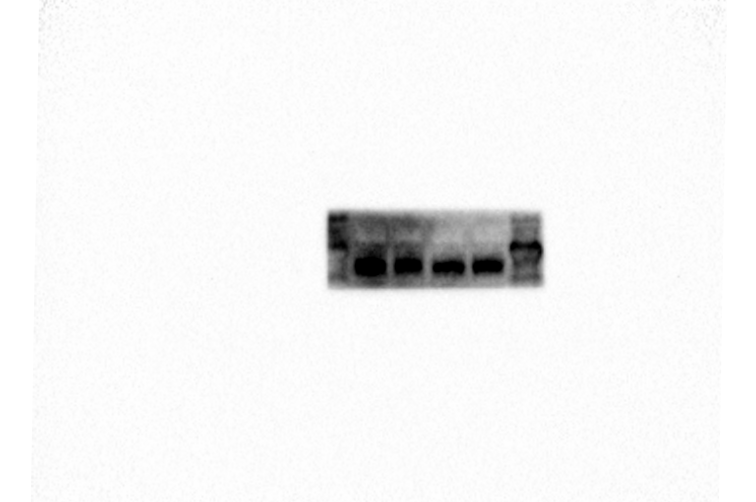


Figure.6 J-CCNB1


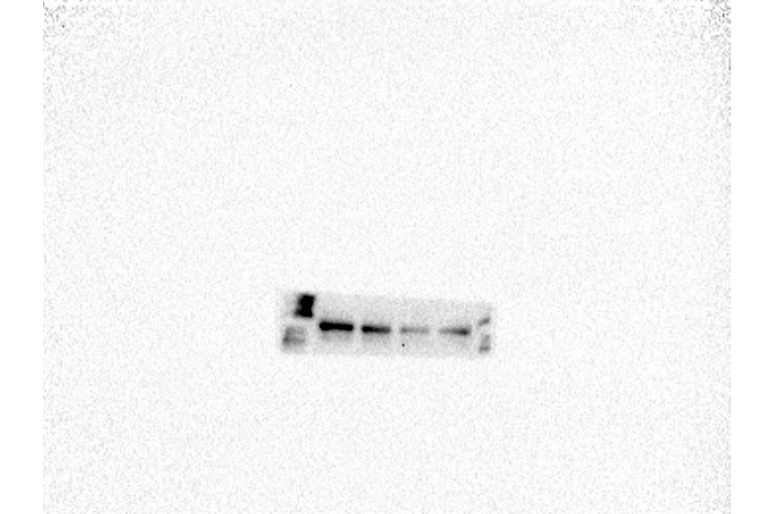


Figure.6 J-CCNE2


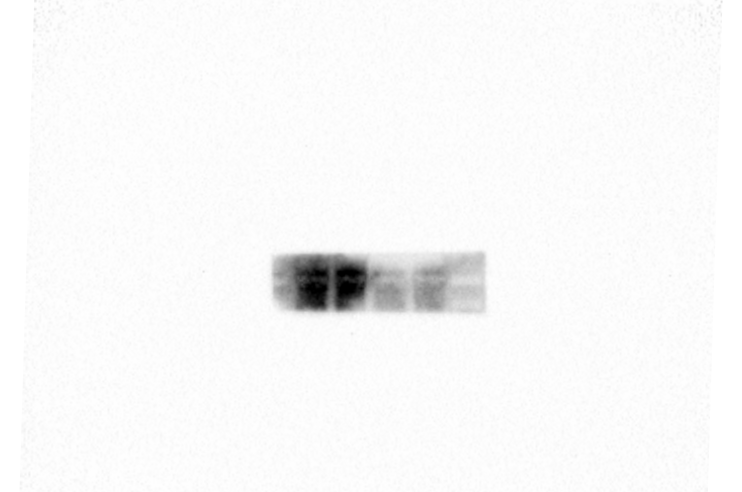


Figure.6 J- β-actin


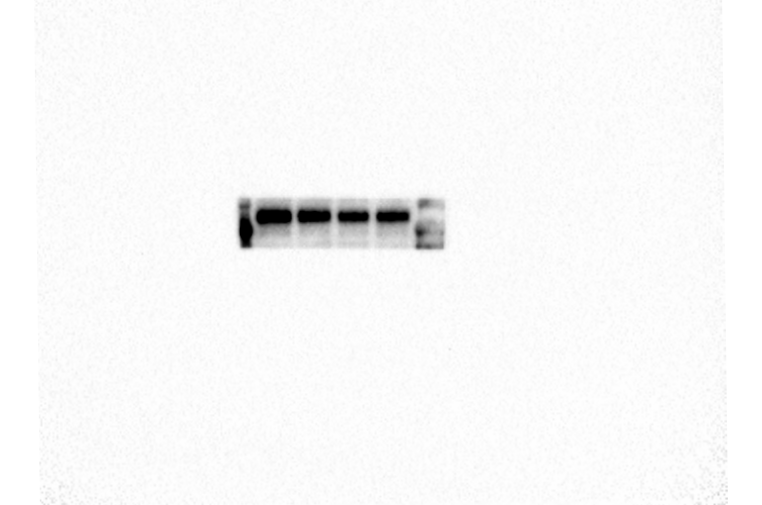


Figure.6 U-PCK1

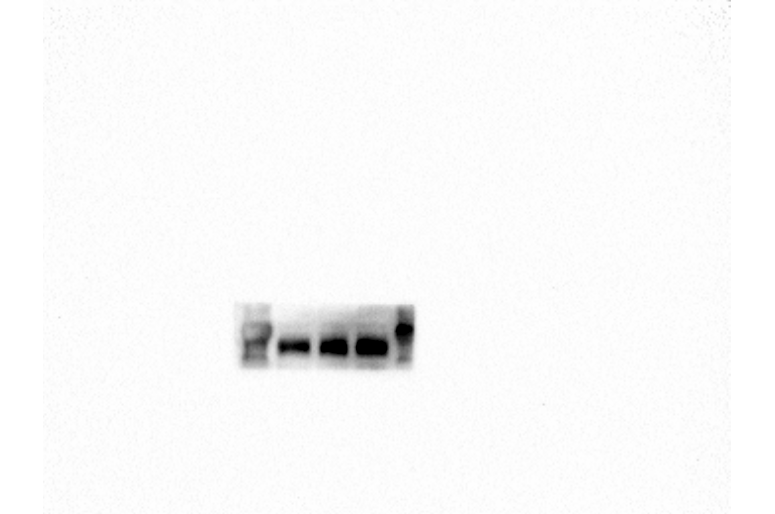


Figure.6 U-CCNB1


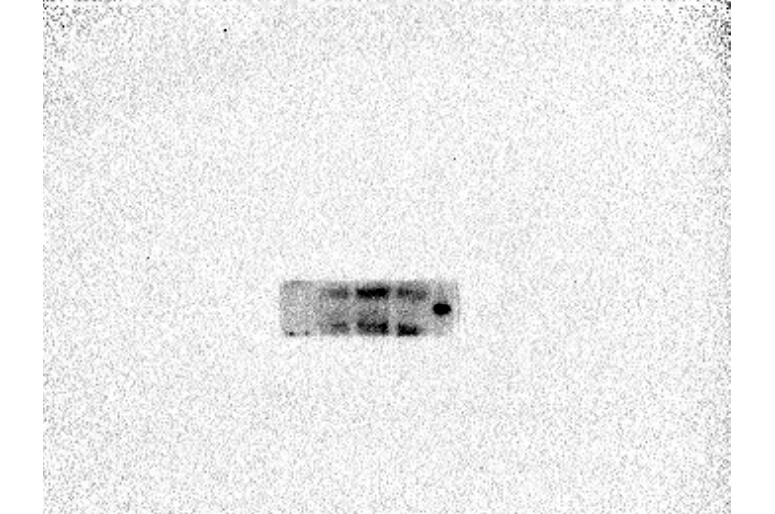


Figure.6 U-CCNE2


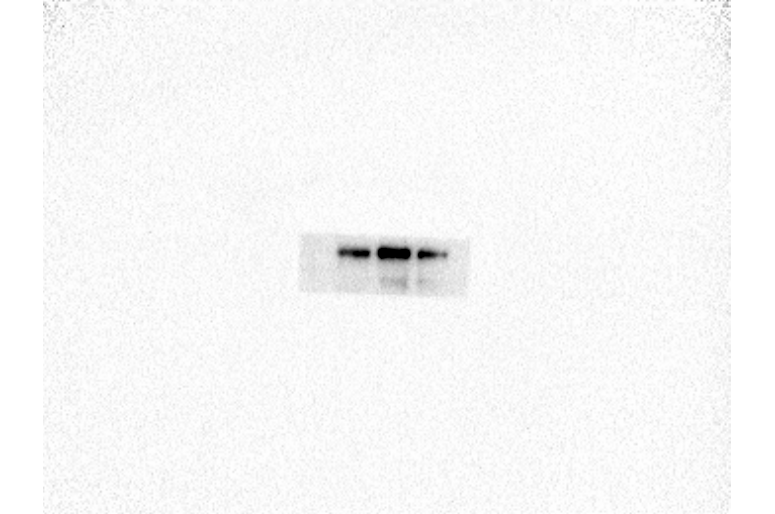


Figure.6 U-β-actin

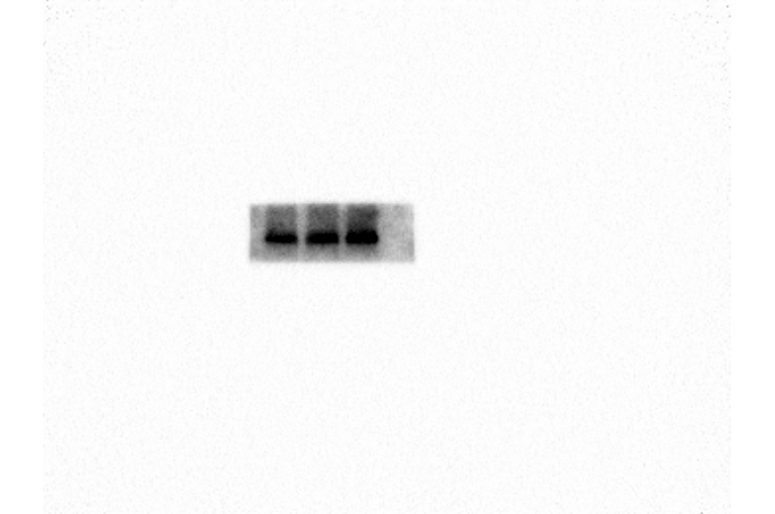


Figure.8 B-PCK1


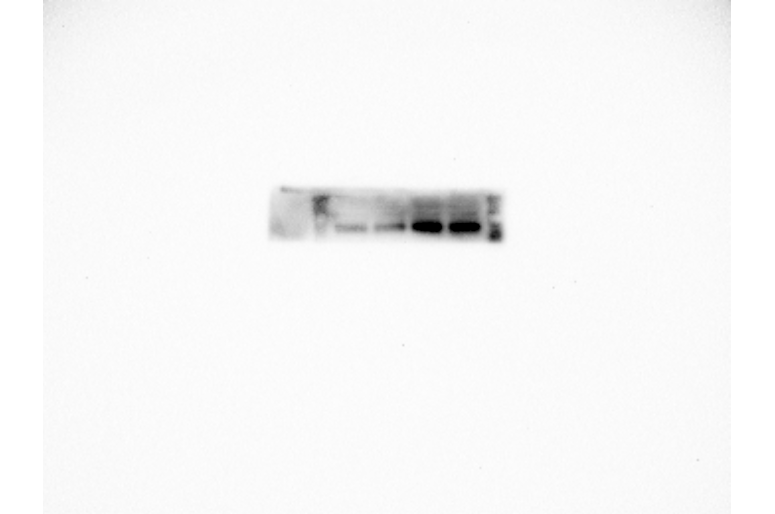


Figure.8 B-CCNB1


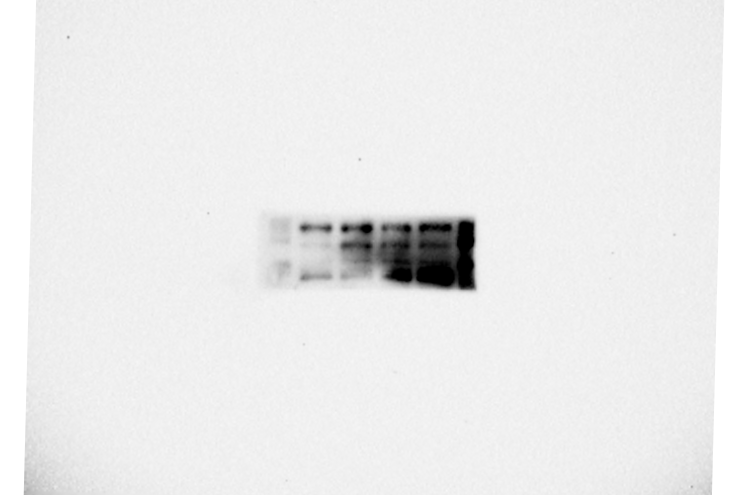


Figure.8 B-CCNE2


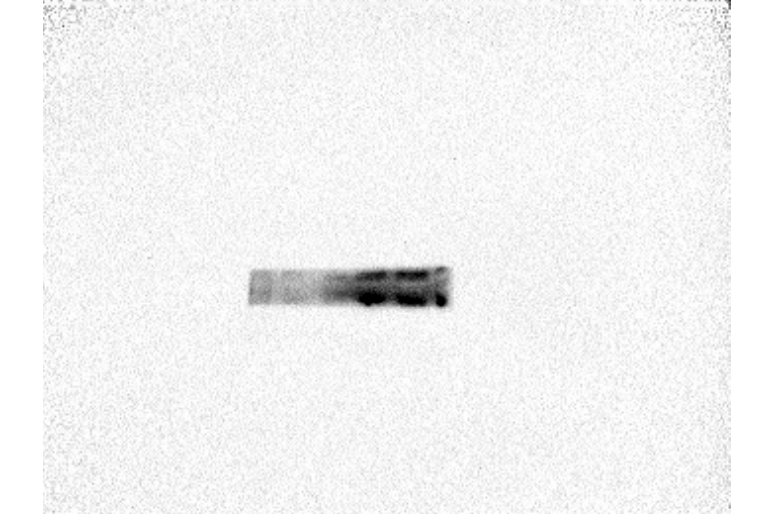


Figure.8 B-β-actin


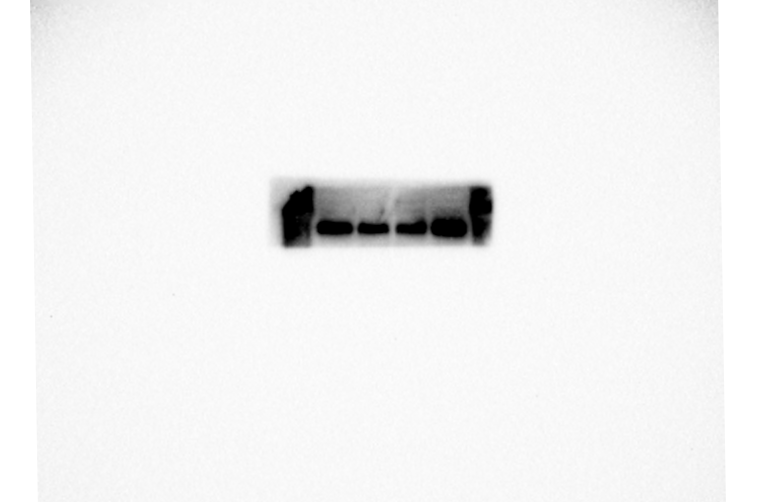


Figure.8 E-PCK1


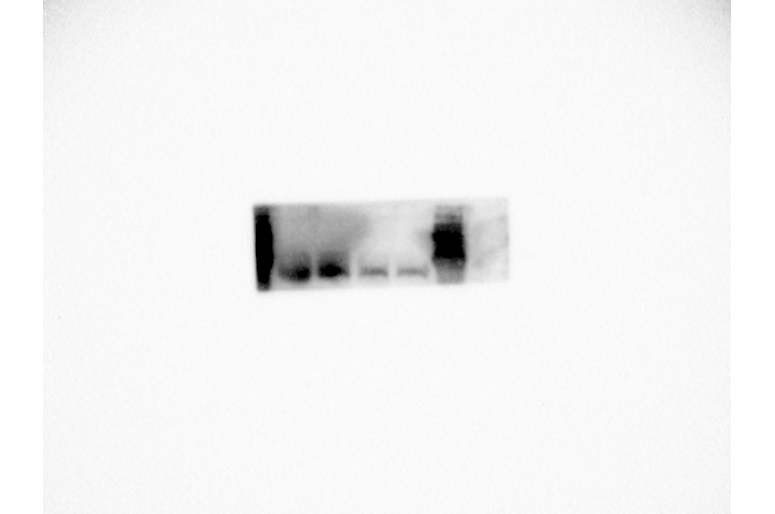


Figure.8 E-CCNB1


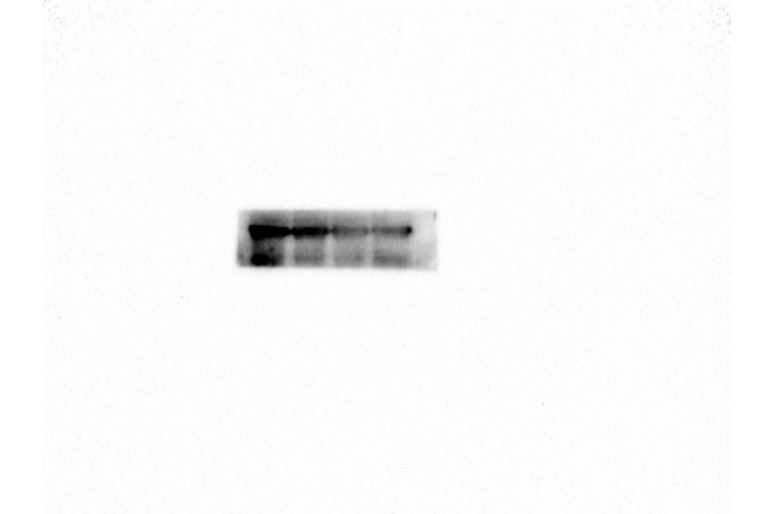


Figure.8 E-CCNE2


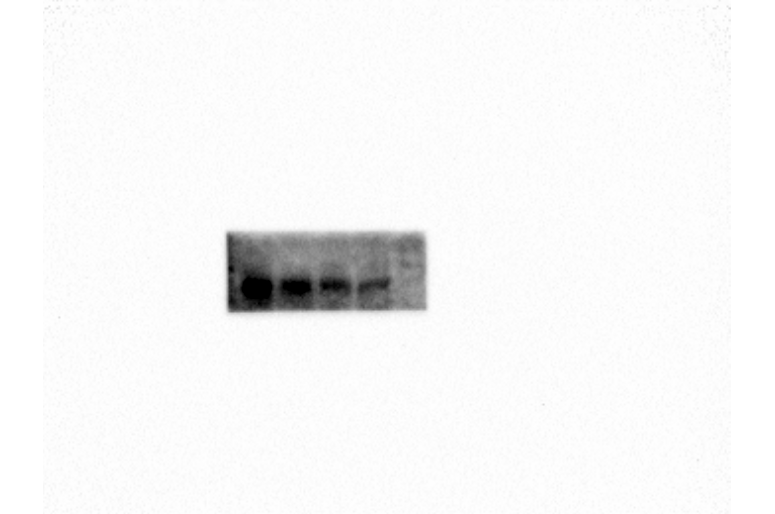


Figure.8 E-β-actin


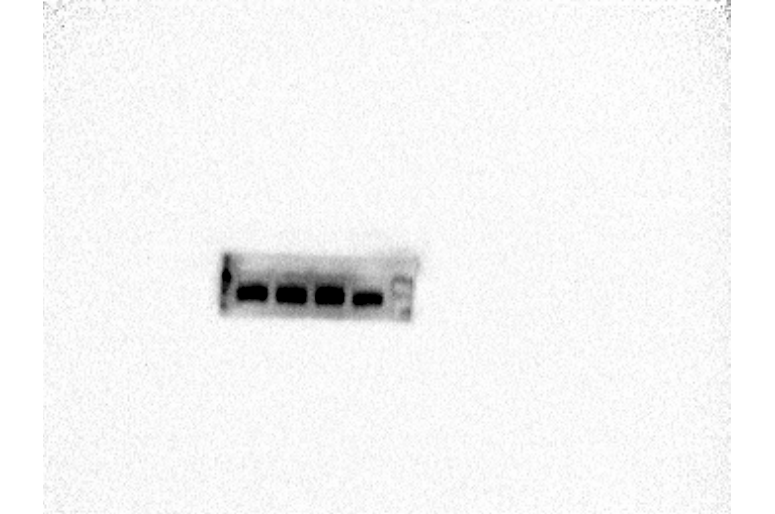


Figure.8 P-PCK1


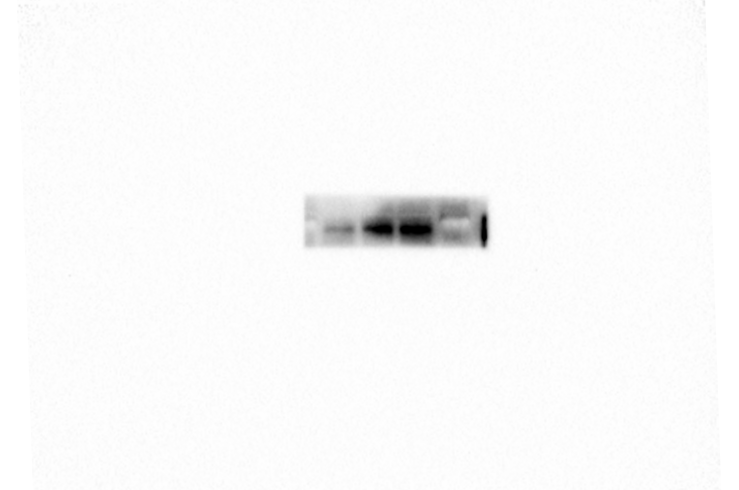


Figure.8 P-CCNB1


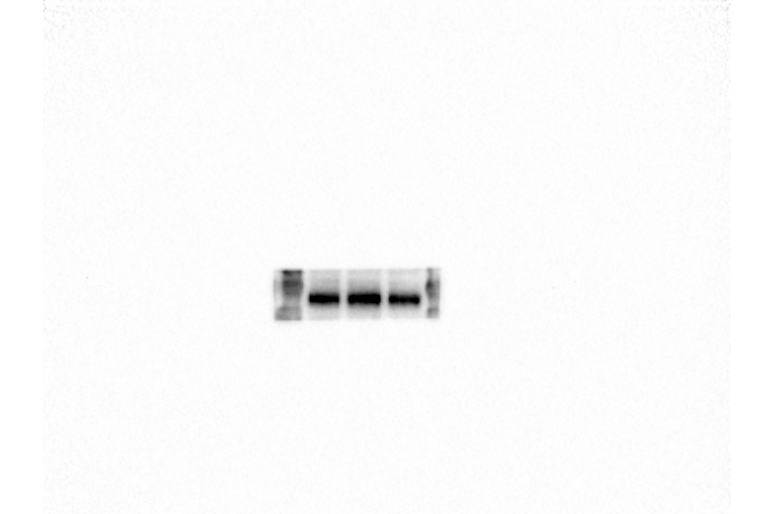


Figure.8 P-CCNE2


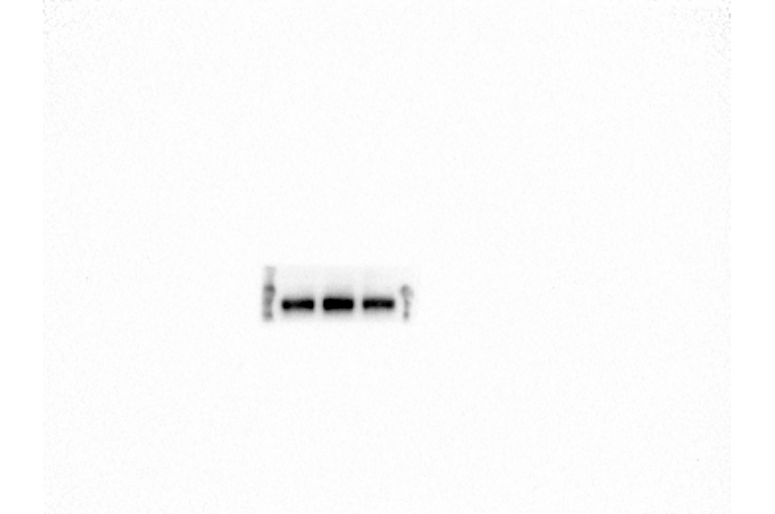


Figure.8 P-β-actin


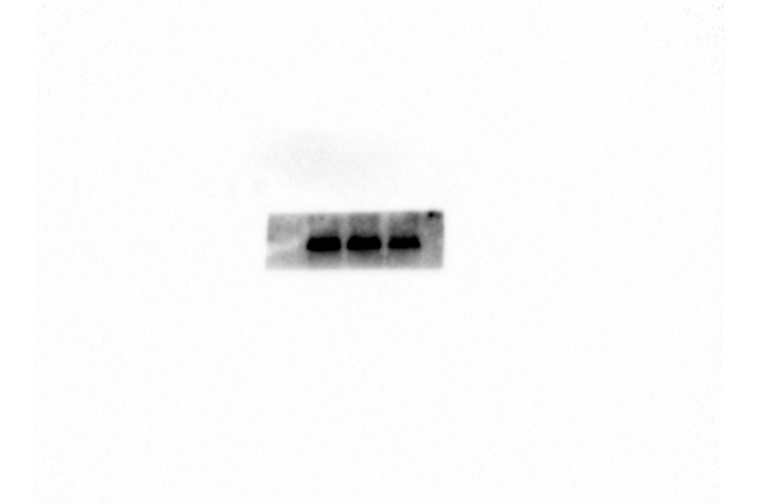

Supplement: Supplementary file 1 — Additional file 1: Table S1. siRNAs used in this study. Table S2. Primers used in qRT-PCR assays. Table S3. Antibody information. Fig. S1. Immunofluorescence identification of testicular Leydig cells. Fig. S2. Upregulated differential genes KEGG pathway enrichment analysis. Fig. S3. Expression level of METTL3 in goat testicular Leydig cells. Fig. S4. miR-145-3p and PCK1 double luciferin analysis. [file 40104_2025_1307_MOESM1_ESM.docx]
